# Atg38-Atg8 interaction in fission yeast establishes a positive feedback loop to promote autophagy

**DOI:** 10.1080/15548627.2020.1713644

**Published:** 2020-01-19

**Authors:** Zhong-Qiu Yu, Ling-Ling Sun, Zhao-Di Jiang, Xiao-Man Liu, Dan Zhao, Hai-Tao Wang, Wan-Zhong He, Meng-Qiu Dong, Li-Lin Du

**Affiliations:** a National Institute of Biological Sciences, Beijing, China; b PTN Graduate Program, School of Life Sciences, Peking University, Beijing, China; c Tsinghua Institute of Multidisciplinary Biomedical Research, Tsinghua University, Beijing, China

**Keywords:** AIM, Atg38, Atg8, autophagy, positive feedback loop, Ptdins3k complex I

## Abstract

Macroautophagy (autophagy) is driven by the coordinated actions of core autophagy-related (Atg) proteins. Atg8, the core Atg protein generally considered acting most downstream, has recently been shown to interact with other core Atg proteins via their Atg8-family-interacting motifs (AIMs). However, the extent, functional consequence, and evolutionary conservation of such interactions remain inadequately understood. Here, we show that, in the fission yeast *Schizosaccharomyces pombe*, Atg38, a subunit of the phosphatidylinositol 3-kinase (PtdIns3K) complex I, interacts with Atg8 via an AIM, which is highly conserved in Atg38 proteins of fission yeast species, but not conserved in Atg38 proteins of other species. This interaction recruits Atg38 to Atg8 on the phagophore assembly site (PAS) and consequently enhances PAS accumulation of the PtdIns3K complex I and Atg proteins acting downstream of the PtdIns3K complex I, including Atg8. The disruption of the Atg38-Atg8 interaction leads to the reduction of autophagosome size and autophagic flux. Remarkably, the loss of this interaction can be compensated by an artificial Atg14-Atg8 interaction. Our findings demonstrate that the Atg38-Atg8 interaction in fission yeast establishes a positive feedback loop between Atg8 and the PtdIns3K complex I to promote efficient autophagosome formation, underscore the prevalence and diversity of AIM-mediated connections within the autophagic machinery, and reveal unforeseen flexibility of such connections.

**Abbreviations**: AIM: Atg8-family-interacting motif; AP-MS: affinity purification coupled with mass spectrometry; Atg: autophagy-related; FLIP: fluorescence loss in photobleaching; PAS: phagophore assembly site; *PB: piggyBac*; PE: phosphatidylethanolamine; PtdIns3K: phosphatidylinositol 3-kinase; PtdIns3P: phosphatidylinositol 3-phosphate.

## Introduction

Macroautophagy (hereafter autophagy) is an evolutionarily conserved degradation pathway in eukaryotes, by which intracellular materials are transported into lysosomes or vacuoles. Autophagy is characterized by the sequestration of cytosol and organelles into double-membraned vesicles called autophagosomes, which are formed by nucleation and subsequent expansion of membrane structures known as phagophores. Autophagy has been linked to many physiological processes including adaptation to stress, cell differentiation and development, aging, neurodegenerative diseases, and cancer [1,2].

The molecular machinery required for autophagosome formation has been most extensively studied in the budding yeast *Saccharomyces cerevisiae* [3–5]. To date, more than 40 autophagy-related (Atg) proteins have been identified in *S. cerevisiae*. A subset of these Atg proteins, referred to as the core Atg proteins, is required for all types of autophagy and plays essential roles in the biogenesis of autophagosomes [5,6]. Core Atg proteins are classified into 6 functional groups: the Atg1 complex, the transmembrane protein Atg9, the autophagy-specific phosphatidylinositol 3-kinase (PtdIns3K) complex I, the Atg2-Atg18 complex, the Atg12 conjugation system, and the Atg8 conjugation system. After autophagy induction, these 6 groups of proteins are recruited to a punctate structure termed the phagophore assembly site (PAS) in a hierarchical manner [3,7–11].

The ubiquitin-like protein Atg8 plays a critical role in phagophore expansion [12,13]. Upon starvation, Atg8 is anchored to the phagophore via its covalent conjugation to the membrane lipid phosphatidylethanolamine (PE). The formation of Atg8–PE requires the sequential actions of a cysteine protease Atg4, E1-like enzyme Atg7, E2-like conjugation enzyme Atg3, and E3-like ligase the Atg12–Atg5-Atg16 complex [14–16]. PE-conjugated Atg8 on autophagic membranes acts as a scaffold to recruit Atg8-interacting proteins to the phagophore. Atg8-interacting proteins bind to Atg8 through a conserved short linear motif called the Atg8-family-interacting motif (AIM) or LC3-interacting region (LIR) [17–20]. The consensus sequence for the core AIM is W/F/YxxL/I/V, which is often surrounded by acidic residues. Structural studies revealed that the 2 conserved residues in the core AIM dock into 2 hydrophobic pockets on Atg8 [20]. Recently, a growing number of core Atg proteins, such as budding yeast Atg1 [21,22], budding yeast Atg3 [23,24], budding yeast Atg4 [25], multiple subunits of mammalian ULK1 complex [21,26–28], mammalian ATG4B [29], and the VPS34, BECN1/Beclin 1, and ATG14 subunits of mammalian class III PtdIns3K complex I [30], have been shown to contain functional AIMs.

In budding yeast, there are 2 PtdIns3K complexes: the PtdIns3K complex I and the PtdIns3K complex II. The former, composed of Vps34, Vps15, Atg6/Vps30, Atg14, and Atg38, functions in autophagy, and the latter, composed of Vps34, Vps15, Atg6, and Vps38, participates in vacuolar protein sorting [31–33]. Atg14 and Vps38, which are complex-specific subunits, are responsible for PAS localization of the PtdIns3K complex I and endosome localization of the PtdIns3K complex II, respectively [32]. The other complex I-specific subunit, Atg38, helps to maintain the integrity of complex I [33]. NRBF2 is an apparent orthologue of budding yeast Atg38 in mammals, but its exact roles remain unresolved [34–39].

The fission yeast *Schizosaccharomyces pombe*, which is evolutionarily distant from the budding yeast *S. cerevisiae*, has become an important model organism for dissecting the mechanisms of autophagy [40–48]. Fission yeast Vps34 was identified in 1995 [49,50], but its binding partners have not been formally characterized. Among its expected binding partners, Vps15 and Atg6 are well conserved, whereas Atg14, Vps38, and Atg38 cannot be recognized by sequence homology alone. In our previous work, we used a genome-wide deletion library to search for fission yeast autophagy genes and identified genes encoding fission yeast Atg14 and Vps38 but did not find a gene encoding Atg38 [40].

In this study, we first identified a previously uncharacterized protein as *S. pombe* Atg38. We showed that it is an important member of the PtdIns3K complex I. Sequence conservation among 4 fission yeast species led to our discovery of an AIM in *S. pombe* Atg38. We revealed that the Atg38-Atg8 interaction establishes a positive feedback loop between Atg8 and the PtdIns3K complex I to facilitate autophagosome formation.

## Results

### Identification of fission yeast Atg38

In the fission yeast *S. pombe*, cells lacking the vacuolar protease gene *isp6* lose viability after nitrogen starvation, and this phenotype can be suppressed by deleting an autophagy gene [42] (**Fig. S1A**). Based on this phenomenon, we searched for autophagy genes by performing an *isp6Δ* suppressor screen using *piggyBac* (*PB*) transposon-based mutagenesis [51] (**Fig. S1B**). *isp6Δ* suppressors uncovered in the screen include *PB* insertions in *atg1, atg2, atg3, atg4, atg5, atg7, atg8, atg9, atg12, atg13, atg16, atg17, atg18b, atg18c*, and *ctl1* (**Fig. S1C**), which are previously characterized autophagy genes [40]. In addition, multiple independent *PB* insertions in *SPBC660.08*, an uncharacterized gene, were isolated in this screen, suggesting that the SPBC660.08 protein may be an autophagy factor.

**Figure 1.**
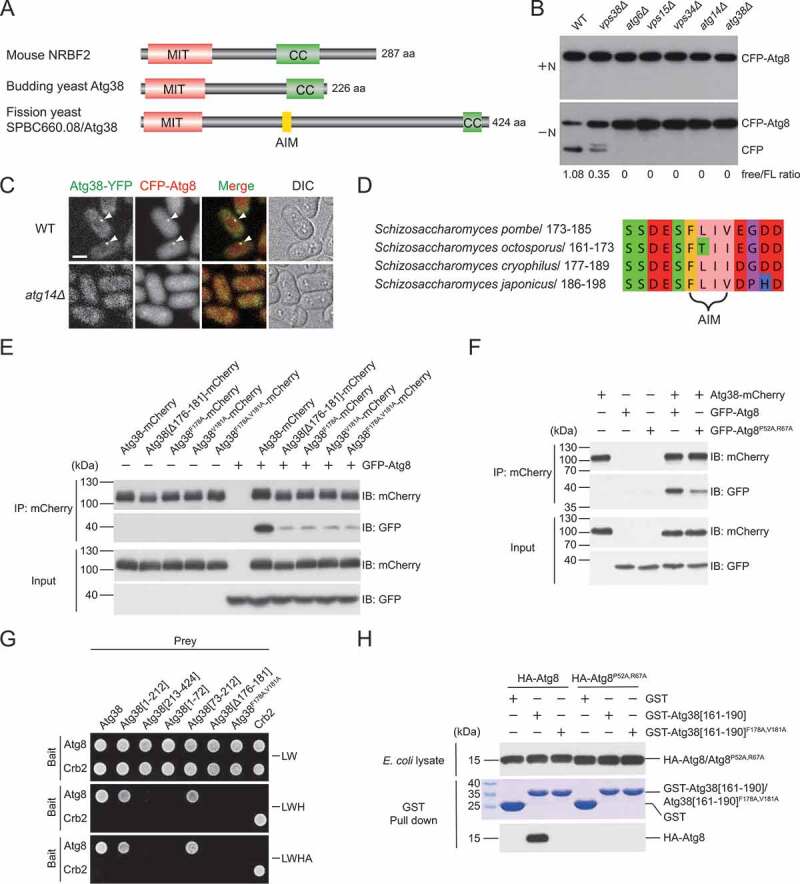
Identification of *S. pombe* Atg38 and its AIM-mediated interaction with Atg8. (A) The domain organization of mouse NRBF2, budding yeast Atg38, and fission yeast SPBC660.08/Atg38. MIT, microtubule interacting and trafficking domain. CC, coiled-coil domain. AIM, Atg8-family-interacting motif. (B) Deletion of *atg38* blocked starvation-induced processing of CFP-Atg8. Cells of wild type (WT) and mutants lacking PtdIns3K complexes subunits were collected before (+N) and after shifting to nitrogen-free medium for 10 h (−N), and the total lysates were analyzed by immunoblotting with antibody against CFP. (C) Atg38 colocalized with Atg8 at cytoplasmic puncta induced by starvation, and *atg14Δ* abolished the puncta of Atg38 and Atg8. Mid-log phase cells expressing YFP-tagged Atg38 and CFP-tagged Atg8 were incubated in nitrogen-free medium for 2 h, and then imaged by fluorescence microscopy. Arrowheads point to representative puncta where Atg38 and Atg8 colocalized. Scale bar: 3 μm. (D) The most conserved region of Atg38 among *S. pombe, S. octosporus, S. cryophilus*, and *S. japonicus*. (E-F) Coimmunoprecipitation between Atg38 and Atg8 was diminished by the AIM mutation in Atg38 (E) and the AIM-binding region mutation in Atg8 (F). Atg8 and Atg8^P52A,R67A^ were tagged with GFP. mCherry-tagged wild-type or AIM-mutated Atg38 was immunoprecipitated with mCherry-trap agarose beads. Total cell lysates and mCherry-trap precipitates were analyzed by immunoblotting with antibody against mCherry or GFP. (G) In yeast two-hybrid assay, Atg38 interacted with Atg8 in a manner dependent on the AIM. (H) An *in vitro* affinity-isolation assay between GST-tagged Atg38 fragments and HA-tagged Atg8 or Atg8^P52A,R67A^. Coomassie Brilliant Blue R-250 (CBB) stained gel shows the GST construct inputs. Western blot was probed with antibody against HA

Coincidentally, we identified SPBC660.08 in a parallel effort of searching for interactors of known autophagy factors using affinity purification coupled with mass spectrometry (AP-MS) analysis. SPBC660.08 was co-purified with Atg6 (**Fig. S1D**), a shared subunit of the PtdIns3K complex I and complex II, and Atg14 (**Fig. S1E**), a complex I-specific subunit, suggesting that it is a subunit of the PtdIns3K complex I. Furthermore, Vps34, Vps15, Atg6, and Atg14, but not Vps38, a complex II-specific subunit, were co-purified with SPBC660.08 (**Fig. S1F**), suggesting that SPBC660.08, like Atg14, is complex I-specific. SPBC660.08 contains an N-terminal MIT domain and a C-terminal coiled-coil domain, and thus shares the same domain organization with budding yeast Atg38 and mammalian NRBF2 (Figure 1A **and S1G**). We postulated that SPBC660.08 is the fission yeast counterpart of budding yeast Atg38 and mammalian NRBF2, and named it Atg38.

We next investigated the phenotypes of *atg38* deletion and the subcellular localization of Atg38. Consistent with the *isp6Δ* suppressor screen results, *atg38Δ* mutant was defective in nitrogen starvation-induced autophagy, indicated by the CFP-Atg8 processing assay, where the accumulation of vacuolar-protease-resistant free CFP serves as a readout of the autophagic flux (Figure 1B). Furthermore, like *atg14Δ* mutant but unlike mutants lacking subunits of the PtdIns3K complex II, *atg38Δ* mutant was not temperature sensitive, had normal-sized vacuoles, and did not mistakenly sort Cpy1 to the cell surface (**Fig. S2**), indicating that Atg38 plays no roles in the functions of the PtdIns3K complex II. As reported before for Atg14 [40], Atg38 formed starvation-induced puncta colocalizing with Atg8, a marker of the PAS (Figure 1C). Deleting *atg14* abolished puncta formed by Atg38 and Atg8. Together, these results indicate that fission yeast Atg38 is a functionally important subunit of the PtdIns3K complex I.

### An AIM in Atg38 directly interacts with Atg8

By aligning the protein sequences of Atg38 proteins from *S. pombe* and 3 other fission yeast species, we noticed that the most conserved region in Atg38 is not the N-terminal MIT domain or the C-terminal coiled-coil domain. Instead, a 13-amino-acid linear motif between residues 173 and 185 is the most conserved region among fission yeast Atg38 proteins (Figure 1A,D). This region contains an aromatic residue Phe178 and a hydrophobic residue Val181 separated by 2 residues and flanked by negatively charged residues, thus bearing all the hallmarks of an AIM.

To test whether this AIM indeed mediates a physical interaction between Atg38 and Atg8, we first performed coimmunoprecipitation experiments. We found that Atg8 coimmunoprecipitated with wild-type Atg38, and this interaction was severely impaired when the central sequence of the AIM was deleted (*Δ176-181*), or when one or both of Phe178 and Val181 were substituted with alanine (Figure 1E). Conversely, when we introduced into Atg8 a pair of point mutations (P52A,R67A) known to weaken AIM-Atg8 interactions in budding yeast [19], we also observed a reduction of the Atg38-Atg8 interaction (Figure 1F). These coimmunoprecipitation data indicate that Atg38 interacts with Atg8, and this interaction is mediated by the AIM in Atg38 and the AIM-binding region in Atg8.

To further examine the Atg38-Atg8 interaction in a heterologous system, we performed yeast two-hybrid (Y2H) assay, which confirmed that Atg38 interacts with Atg8 in an AIM-dependent manner, and showed that a 140-amino-acid region downstream of the MIT domain, Atg38 [73-212], is sufficient for binding Atg8 (Figure 1G). We then expressed in *Escherichia coli* HA-tagged Atg8 and a GST-tagged 30-amino-acid Atg38 fragment, GST-Atg38[161–190], which encompasses the AIM, and performed affinity-isolation experiments. GST-Atg38[161–190], but not GST alone, efficiently pulled down Atg8 (Figure 1H). Either mutating Phe178 and Val181 in Atg38[161–190] or mutating Pro52 and Arg67 in Atg8 blocked the Atg38-Atg8 interaction. Together, these results demonstrate that Atg38 directly interacts with Atg8 via the AIM.

Hereafter we will refer to Atg38^F178A,V181A^ mutations as Atg38[AIM mut] and refer to Atg8^P52A,R67A^ mutations as Atg8[AB mut] (for AIM-binding defective). They were used for dissecting the *in vivo* function of the Atg38-Atg8 interaction.

### The Atg38-Atg8 interaction promotes autophagy

**Figure 2.**
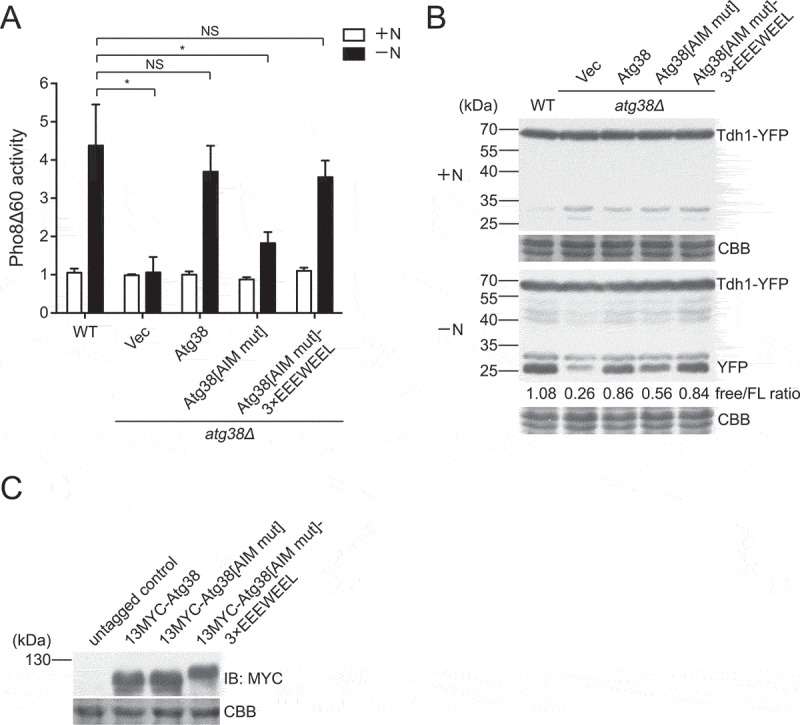
The Atg38 AIM promotes autophagy by interacting with Atg8. (A) Autophagic flux measurement using the Pho8Δ60 assay was performed in wild-type cells and *atg38Δ* cells transformed with an empty vector or a plasmid expressing wild-type Atg38, Atg38[AIM mut], or Atg38[AIM mut] inserted with an exogenous AIM (3× EEEWEEL) between Ala181 and Glu182. Cells were collected before (+N) and after shifting to nitrogen-free medium for 4 h (−N). Average activity from non-starved samples was set to 1. Data are mean ± s.d. of triplicates from representative experiments. * indicates *P* < 0.05; NS, not significant. *P* values were calculated using Welch’s t-test. (B) Starvation-induced processing of Tdh1-YFP was examined in wild-type cells and *atg38Δ* cells transformed with an empty vector or a plasmid expressing wild-type Atg38, Atg38[AIM mut], or Atg38[AIM mut] inserted with an exogenous AIM between Ala181 and Glu182. Cells expressing YFP-tagged Tdh1 were collected before (+N) and after shifting to nitrogen-free medium for 2 h (−N), and the total lysates were analyzed by immunoblotting with antibody against YFP. Coomassie Brilliant Blue R-250 (CBB) staining of PVDF membrane after immunodetection served as protein loading control. Note that faint bands around 25–35 kDa in +N medium may result from the degradation of Tdh1-YFP by proteasome or may occur in the process of cell lysis. (C) Cells carrying the indicated Atg38 constructs and an untagged control were collected after 2 h of starvation, and then analyzed by immunoblotting with antibody against MYC. Coomassie Brilliant Blue R-250 (CBB) staining of PVDF membrane after immunodetection served as protein loading control

To investigate whether the Atg38-Atg8 interaction functions in autophagy, we used 2 different assays to analyze the effect of the Atg38 AIM mutation on autophagy activity. First, we adopted the Pho8Δ60 assay, a well-established autophagy assay in budding yeast [52], by expressing *S. cerevisiae* Pho8Δ60 in an *S. pombe pho8Δ* strain. In this assay, Pho8Δ60 is transported into the vacuole and subsequently activated in an autophagy-dependent manner. Thus, the alkaline phosphatase activity of Pho8Δ60 can be used as a quantitative readout of autophagy [52]. In wild-type cells, the alkaline phosphatase activity of Pho8Δ60 increased in response to starvation, whereas in *atg38Δ* cells, no elevation of Pho8Δ60 activity was observed (Figure 2A), consistent with the result of the CFP-Atg8 processing assay shown earlier. Reintroducing Atg38 in *atg38Δ* cells restored post-starvation Pho8Δ60 activity to the wild-type level. In contrast, Pho8Δ60 activity was only restored to about half of the wild-type level when expressing Atg38[AIM mut].

Second, we used the Tdh1-YFP processing assay to monitor autophagy. Tdh1 is the major form of fission yeast GAPDH (glyceraldehyde-3-phosphate dehydrogenase), and the processing of Tdh1-YFP to free YFP has been used as a readout for autophagy [41]. Consistent with the result of the Pho8Δ60 assay, starvation in wild-type cells generated free YFP, but not in *atg38Δ* cells, and the amount of free YFP generated in *atg38Δ* cells expressing Atg38[AIM mut] was about half of that in wild-type cells or *atg38Δ* cells expressing Atg38 (Figure 2B). We compared the protein levels of AIM-mutated Atg38 and wild-type Atg38 and found no difference (Figure 2C). Together, the results of these 2 assays indicate that the Atg38 AIM has an important role in promoting autophagy.

To rule out the possibility that the Atg38 AIM may promote autophagy through mechanism(s) other than interacting with Atg8, we performed a rescue experiment. If binding Atg8 is the main function of the Atg38 AIM in autophagy, it might be possible to rescue the defects of Atg38[AIM mut] with an exogenous AIM. To this end, we used a 3×EEEWEEL motif based on the EEEWEEL sequence, which is an artificially designed AIM sequence [53]. Inserting the 3×EEEWEEL immediately downstream of the mutated core AIM in Atg38[AIM mut] restored autophagy activity to the wild-type level (Figure 2A,B). The protein level of Atg38[AIM mut]-3×EEEWEEL was not higher than Atg38[AIM mut] (Figure 2C). Therefore, we concluded that the main function of the Atg38 AIM in autophagy is mediating interaction with Atg8.

Since Atg38-Atg8 interaction requires both the AIM in Atg38 and the AIM-binding region in Atg8, we expected that Atg8[AB mut] also has a defect in autophagy. Indeed, we observed a decrease of starvation-induced autophagy activity in *atg8Δ* cells expressing Atg8[AB mut], shown by the Pho8Δ60 assay (**Fig. S3A**). However, we cannot exclude the possibility that the reduced ability of Atg8[AB mut] to interact with protein(s) other than Atg38 also contributes to its autophagy defect. Collectively, these results establish that Atg38-Atg8 interaction is important for autophagy.

### The Atg38 AIM mutation diminishes PAS accumulation of the PtdIns3K complex I and of Atg proteins acting downstream of the PtdIns3K complex I

**Figure 3.**
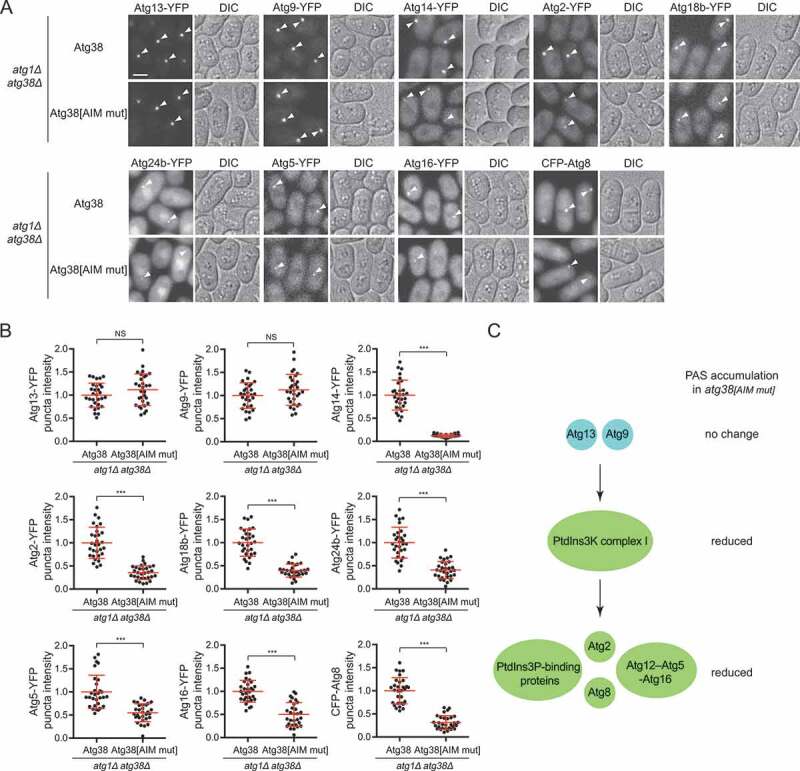
The AIM mutation in Atg38 influences PAS accumulation of the PtdIns3K complex I and of Atg proteins downstream of the PtdIns3K complex I, but not of Atg proteins upstream of the PtdIns3K complex I. (A) Puncta formation by Atg proteins at the PAS in *atg38Δ* cells expressing Atg38 or Atg38[AIM mut]. Mid-log phase cells expressing YFP or CFP-tagged Atg proteins were incubated in nitrogen-free medium for 2 h, and then imaged by fluorescence microscopy. Arrowheads point to representative puncta formed by Atg proteins. Scale bar: 3 μm. (B) Quantification of the puncta intensity of YFP or CFP-tagged Atg proteins in (A). Mean ± s.d. are shown in red (n = 30). *** indicates *P* < 0.001; NS, not significant. *P* values were calculated using Welch’s t-test. (C) Summary of the effects of the Atg38 AIM mutation on the PAS accumulation of Atg proteins

To explore the mechanism underlying the autophagy-promoting function of the Atg38-Atg8 interaction, we surveyed the influence of the Atg38 AIM mutation on PAS localization of Atg proteins. We first used the *atg1Δ* background, in which Atg proteins accumulate at the PAS [9,40]. The AIM mutation in Atg38 did not affect the starvation-induced PAS accumulation of Atg13 and Atg9 but reduced the PAS accumulation of the PtdIns3K complex I subunit Atg14, phosphatidylinositol 3-phosphate (PtdIns3P)-binding proteins Atg18b and Atg24b, Atg2, and conjugation systems proteins Atg5, Atg16, and Atg8 (Figure 3A,B). In the wild-type background, AIM mutation in Atg38 led to a similar change of PAS accumulation of these Atg proteins as the situation in *atg1Δ* background (**Fig. S4A**). Interestingly, proteins unaffected by the Atg38 AIM mutation are those acting upstream of the PtdIns3K complex I [8,10]. Whereas proteins affected by the Atg38 AIM mutation are those acting downstream of the PtdIns3K complex I or in the case of Atg14, is part of the PtdIns3K complex I (Figure 3C). Furthermore, inserting the 3×EEEWEEL in Atg38[AIM mut] recovered PAS accumulation of the Atg proteins reduced by the Atg38 AIM mutation (**Fig. S4B**). Thus, the Atg38-Atg8 interaction is important for PAS accumulation of the PtdIns3K complex I and of Atg proteins acting downstream of the PtdIns3K complex I, but not of Atg proteins acting upstream of the PtdIns3K complex I.

### The Atg38 AIM mutation reduces the size of autophagosomes

**Figure 4.**
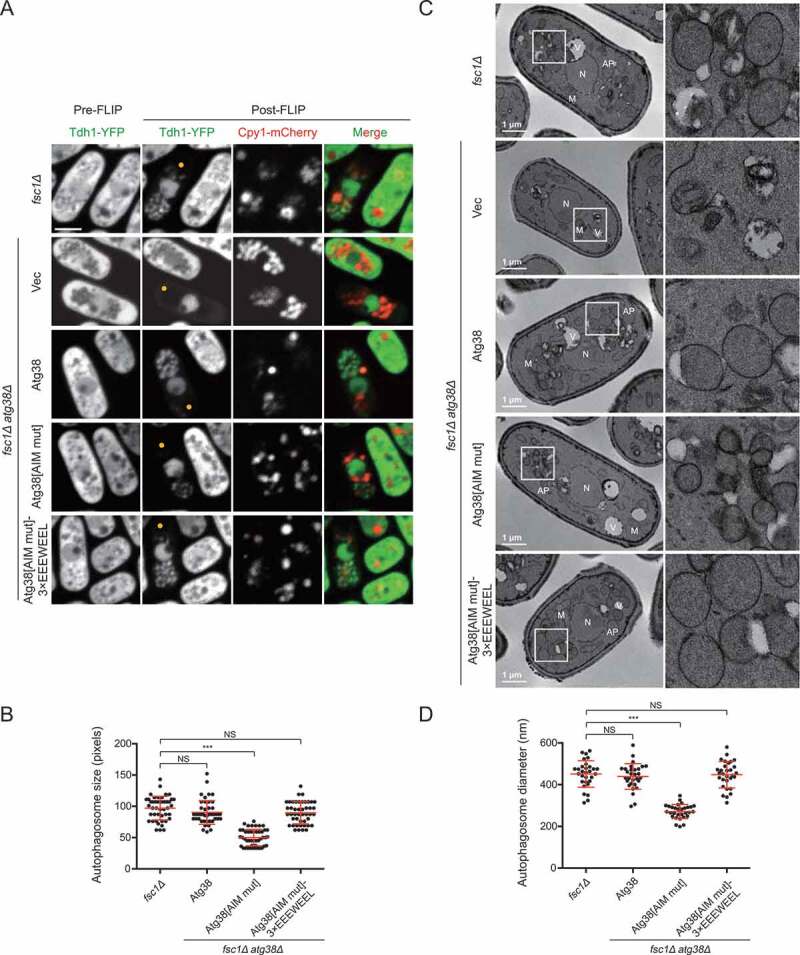
The AIM mutation in Atg38 reduces autophagosome size. (A) Representative images from the FLIP assay of *fsc1Δ* cells and *fsc1Δ atg38Δ* cells carrying an empty vector or the indicated Atg38 constructs. Autophagosomes were rendered visible by fluorescence loss in photobleaching (FLIP) that abolished the diffusible cytoplasmic fluorescent signal of Tdh1-YFP. Aside from Tdh1-YFP trapped inside autophagosomes, a nuclear pool of Tdh1-YFP also remained visible post-FLIP. Cells expressing Tdh1-YFP were collected after 3 h of starvation, and then the FLIP assay was performed. Yellow dots mark the sites of photobleaching. Scale bar: 3 μm. (B) Quantification of the size of autophagosomes in (A). Mean ± s.d. are shown in red (n = 45). *** indicates *P* < 0.001; NS, not significant. *P* values were calculated using Welch’s t-test. (C) Representative electron microscopy images of *fsc1Δ* cells and *fsc1Δ atg38Δ* cells carrying an empty vector or the indicated Atg38 constructs. Cells were collected after 3 h of starvation. N, nucleus; M, mitochondrion; V, vacuole; AP, autophagosome. White squares enclosed regions are shown at higher magnification on the right. (D) Quantification of the diameter of autophagosomes in (C). Mean ± s.d. are shown in red (n = 30). *** indicates *P* < 0.001; NS, not significant. *P* values were calculated using Welch’s t-test

In budding yeast, it was reported that the amount of Atg8 at the PAS determines the size of autophagosomes [13]. Given that the Atg38 AIM mutation diminishes PAS accumulation of many Atg proteins, including Atg8, we examined the size of autophagosomes in *atg38Δ* cells expressing Atg38[AIM mut]. We used the *fsc1Δ* background, in which autophagosomes accumulate due to a block of autophagosome-vacuole fusion [40]. The first method we used to visualize autophagosomes is the fluorescence loss in photobleaching (FLIP) assay, where the cytoplasmic fluorescence signal of Tdh1-YFP is photobleached unless enclosed within autophagosomes [40,41]. Accumulated autophagosomes were observed in *fsc1Δ* cells, but not in *fsc1Δ atg38Δ* cells (Figure 4A), confirming a key role of Atg38 in autophagosome formation. In the *fsc1Δ atg38Δ* background, the expression of wild-type Atg38 resulted in the appearance of normal-sized autophagosomes, whereas the expression of Atg38[AIM mut] resulted in the appearance of autophagosomes with markedly reduced size (Figure 4A,B). A reduction of autophagosome size was also observed in *fsc1Δ atg8Δ* cells expressing Atg8[AB mut] (**Fig. S3B and S3C**).

Because the spatial resolution of fluorescence microscopy is limited, to precisely measure autophagosome size, we further performed transmission electron microscopy (TEM) analysis. A notable size difference was observed between the autophagosomes that accumulated in *fsc1Δ* cells and those that accumulated in *fsc1Δ atg38Δ* cells expressing Atg38[AIM mut] (Figure 4C,D). The average diameter of autophagosomes in *fsc1Δ* cells was 451 nm, whereas the average diameter of autophagosomes in *fsc1Δ atg38Δ* cells expressing Atg38[AIM mut] was 271 nm. Furthermore, in the *fsc1Δ atg38Δ* cells, expressing Atg38[AIM mut]-3×EEEWEEL resulted in the formation of normal-sized autophagosomes (Figure 4). These results show that forming autophagosomes of normal size requires the Atg38 AIM. It is likely that the reduction of autophagosome size is at least partially responsible for the autophagic flux decrease caused by the Atg38 AIM mutation.

### The Atg38-Atg8 interaction promotes PAS accumulation of Atg38

**Figure 5.**
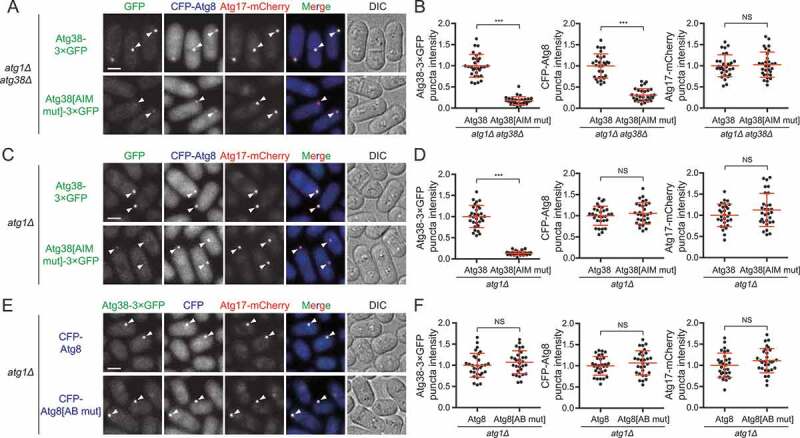
The Atg38-Atg8 interaction functions to directly enhance PAS accumulation of Atg38. (A) The AIM mutation in Atg38 reduced the accumulation of Atg38 and Atg8 at the PAS. Mid-log phase cells expressing 3×GFP-tagged Atg38 or Atg38[AIM mut], CFP-tagged Atg8, and mCherry-tagged Atg17 were incubated in nitrogen-free medium for 2 h, and then imaged by fluorescence microscopy. Arrowheads point to representative puncta where Atg38/Atg38[AIM mut], Atg8, and Atg17 colocalized. (B) Quantification of the puncta intensity of Atg38/Atg38[AIM mut]-3×GFP, CFP-Atg8, and Atg17-mCherry in (A). Mean ± s.d. are shown in red (n = 30). *** indicates *P* < 0.001; NS, not significant. *P* values were calculated using Welch’s t-test. (C) In the presence of endogenous Atg38, the AIM mutation in exogenous Atg38 reduced its PAS accumulation. Mid-log phase cells expressing 3×GFP-tagged Atg38 or Atg38[AIM mut], CFP-tagged Atg8, and mCherry-tagged Atg17 were incubated in nitrogen-free medium for 2 h, and then imaged by fluorescence microscopy. Arrowheads point to representative puncta where Atg38/Atg38[AIM mut], Atg8, and Atg17 colocalized. (D) Quantification of the puncta intensity of Atg38/Atg38[AIM mut]-3×GFP, CFP-Atg8, and Atg17-mCherry in (C). Mean ± s.d. are shown in red (n = 30). *** indicates *P* < 0.001; NS, not significant. *P* values were calculated using Welch’s t-test. (E) In the presence of endogenous Atg8, the AIM-binding region mutation in exogenous Atg8 did not affect its PAS accumulation. Mid-log phase cells expressing 3×GFP-tagged Atg38, CFP-tagged wild-type or AIM-binding defective Atg8, and mCherry-tagged Atg17 were incubated in nitrogen-free medium for 2 h, and then imaged by fluorescence microscopy. Arrowheads point to representative puncta where Atg38, Atg8/Atg8[AB mut], and Atg17 colocalized. (F) Quantification of the puncta intensity of Atg38-3×GFP, CFP-Atg8/Atg8[AB mut], and Atg17-mCherry in (E). Mean ± s.d. are shown in red (n = 30). NS, not significant. *P* values were calculated using Welch’s t-test. Scale bars: 3 μm

When Atg38[AIM mut] was expressed as the only form of Atg38 in the cell at a level equal to that of wild-type Atg38 (**Fig. S5A**), its PAS accumulation in the *atg1Δ* background was substantially lower than that of wild-type Atg38 (Figure 5A,B), suggesting that the primary defect caused by the loss of the Atg38-Atg8 interaction is reduced PAS accumulation of Atg38. However, the PAS accumulation of Atg8 was also reduced by the Atg38 AIM mutation (Figure 5A,B), making it difficult to ascertain whether the primary defect is in PAS accumulation of Atg38 or PAS accumulation of Atg8. To clarify this point, we investigated PAS localization of exogenously expressed Atg38[AIM mut] in the presence of endogenous Atg38. In such a situation, PAS accumulation of Atg8 remained normal (Figure 5C,D). However, PAS accumulation of Atg38[AIM mut] was markedly lower than the wild-type level. We then examined PAS localization of exogenously expressed Atg8[AB mut] in the presence of endogenous Atg8 and found that PAS accumulation of Atg8[AB mut] was normal (Figure 5E,F and **S5B**), suggesting that the Atg38-Atg8 interaction does not directly contribute to PAS accumulation of Atg8. These results indicate that the primary function of the Atg38-Atg8 interaction is to enhance PAS accumulation of Atg38 directly, but not that of Atg8.

Furthermore, we examined whether Atg38-Atg8 interaction facilitates the lipidation of Atg8. Atg38 AIM mutation partially impaired the lipidation of Atg8 (**Fig. S5C, lanes 1 and 4**), and this impairment was recovered by introducing a wild-type *atg38* gene (**lane 5**), suggesting that the Atg38-Atg8 interaction facilitates the lipidation of Atg8. Similarly, in *atg8Δ* cells expressing Atg8[AB mut], the lipidation of Atg8[AB mut] was partially reduced (**lane 6**). However, the reduced lipidation of Atg8[AB mut] cannot be recovered by introducing a wild-type copy of the *atg8* gene (**lane 7**). We reasoned that wild-type Atg8 might compete with Atg8[AB mut] and thus lead to the insufficient lipidation of Atg8[AB mut], and this is supported by the observation that the lipidation of wild-type Atg8 was also reduced when introducing another copy of wild-type *atg8* gene (**lanes 1 and 8**). In the presence of this additional copy of the wild-type *atg8* gene, the lipidation level of Atg8[AB mut] was not lower than Atg8 (**lanes 7 and 8**), suggesting that the Atg38-Atg8 interaction does not directly enhance the lipidation of Atg8.

### The Atg38-Atg8 interaction establishes a positive feedback loop between Atg8 and the PtdIns3K complex I

Core Atg proteins are thought to be recruited to the PAS in a hierarchical manner, in which the PtdIns3K complex I is upstream of Atg8 [3,8–11]. According to this model, PAS accumulation of the PtdIns3K complex I should be independent of Atg8. However, our results above clearly show that through the Atg38-Atg8 interaction Atg8 directly contributes to PAS accumulation of Atg38 (Figure 5C,D), a subunit of the PtdIns3K complex I. We thus proposed that the Atg38-Atg8 interaction establishes a positive feedback loop between Atg8 and the PtdIns3K complex I. This model predicts that the loss of any Atg protein downstream of the PtdIns3K complex I but upstream of Atg8 would reduce PAS accumulation of the PtdIns3K complex I.

**Figure 6.**
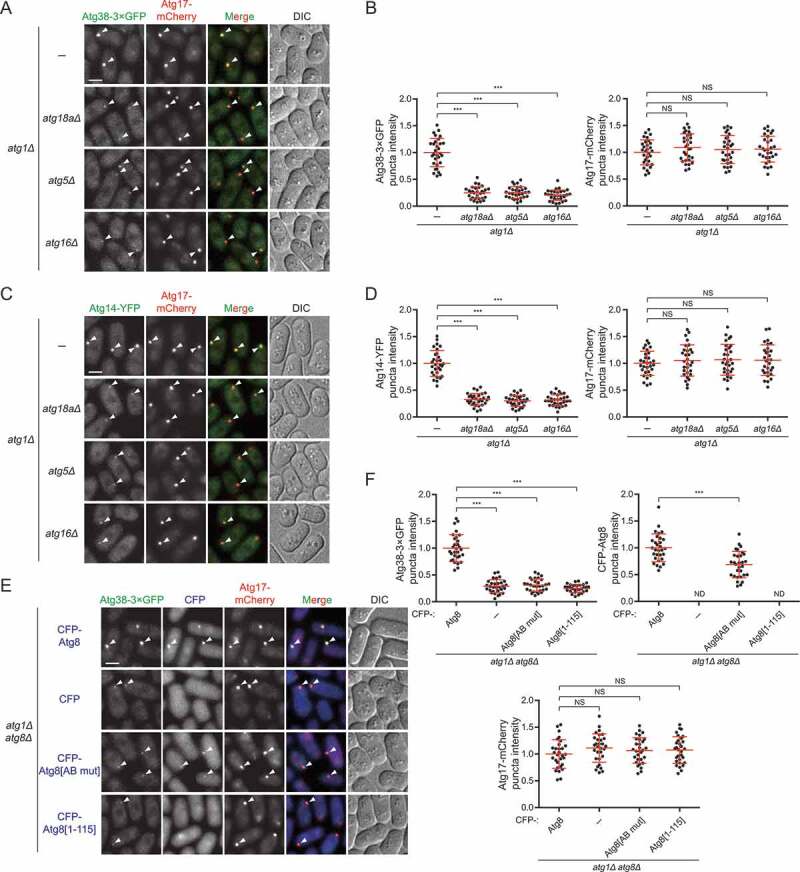
Atg proteins downstream of the PtdIns3K complex I are required for efficient PAS accumulation of the PtdIns3K complex I. (A) PAS accumulation of Atg38 was reduced in the absence of Atg18a, Atg5, or Atg16. Mid-log phase cells expressing 3×GFP-tagged Atg38 and mCherry-tagged Atg17 were incubated in nitrogen-free medium for 2 h, and then imaged by fluorescence microscopy. Arrowheads point to representative puncta where Atg38 and Atg17 colocalized. (B) Quantification of the puncta intensity of Atg38-3×GFP and Atg17-mCherry in (A). Mean ± s.d. are shown in red (n = 30). *** indicates *P* < 0.001; NS, not significant. *P* values were calculated using Welch’s t-test. (C) PAS accumulation of Atg14 was reduced in the absence of Atg18a, Atg5, or Atg16. Mid-log phase cells expressing YFP-tagged Atg14 and mCherry-tagged Atg17 were incubated in nitrogen-free medium for 2 h, and then imaged by fluorescence microscopy. Arrowheads point to representative puncta where Atg14 and Atg17 colocalized. (D) Quantification of the puncta intensity of Atg14-YFP and Atg17-mCherry in (C). Mean ± s.d. are shown in red (n = 30). *** indicates *P* < 0.001; NS, not significant. *P* values were calculated using Welch’s t-test. (E) PAS accumulation of Atg38 was reduced in *atg8Δ* cells, or *atg8Δ* cells expressing an Atg38-interaction-deficient Atg8 or a PE-conjugation-defective Atg8. Mid-log phase cells expressing 3×GFP-tagged Atg38, mCherry-tagged Atg17, and free CFP or CFP-tagged Atg8, Atg8[AB mut], or Atg8[1–115] were incubated in nitrogen-free medium for 2 h, and then imaged by fluorescence microscopy. Arrowheads point to representative puncta where Atg38 and Atg17 (and Atg8) colocalized. (F) Quantification of the puncta intensity of Atg38-3×GFP, CFP-Atg8 and Atg17-mCherry in (E). Mean ± s.d. are shown in red (n = 30). *** indicates *P* < 0.001; NS, not significant; ND, not determined. *P* values were calculated using Welch’s t-test. Scale bars: 3 μm

To test this, using the *atg1Δ* background, we first examined PAS accumulation of Atg38 and Atg14 in the absence of Atg18a, Atg5, or Atg16, which are *S. pombe* autophagy factors acting downstream of the PtdIns3K complex I but upstream of Atg8 [40]. As predicted by the positive feedback loop model, the loss of Atg18a, Atg5, or Atg16 led to a significant decrease in the intensity of puncta formed by Atg38 and Atg14 at the PAS (Figure 6A**–**D). The decreased intensity of puncta formed by Atg38 and Atg14 at the PAS was not due to the reduced protein levels, for the loss of Atg18a, Atg5, or Atg16 did not influence the protein levels of Atg38 and Atg14 (**Fig. S6**). These results show that Atg proteins downstream of the PtdIns3K complex I contribute to normal PAS accumulation of the PtdIns3K complex I and thus lend support to the model that a positive feedback loop exists between Atg8 and the PtdIns3K complex I.

Next, we examined the PAS accumulation of Atg38 in the absence of the most downstream Atg factor, Atg8. Consistent with the situation in mutants lacking Atg18a, Atg5, or Atg16, we observed a pronounced decrease of PAS accumulation of Atg38 in *atg8Δ* cells (Figure 6E,F). In addition, we found that neither the Atg38-interaction-deficient Atg8 (Atg8[AB mut]), nor the PE-conjugation-defective Atg8 (Atg8[1–115]), which cannot localize to the PAS but should still be capable of binding Atg38, could complement the diminished PAS accumulation of Atg38 caused by *atg8Δ* (Figure 6E,F), demonstrating that the positive feedback loop between Atg8 and the PtdIns3K complex I is established by Atg38 interacting with Atg8 on the PAS.

### The loss of the Atg38-Atg8 interaction can be compensated by an artificial Atg14-Atg8 interaction

**Figure 7.**
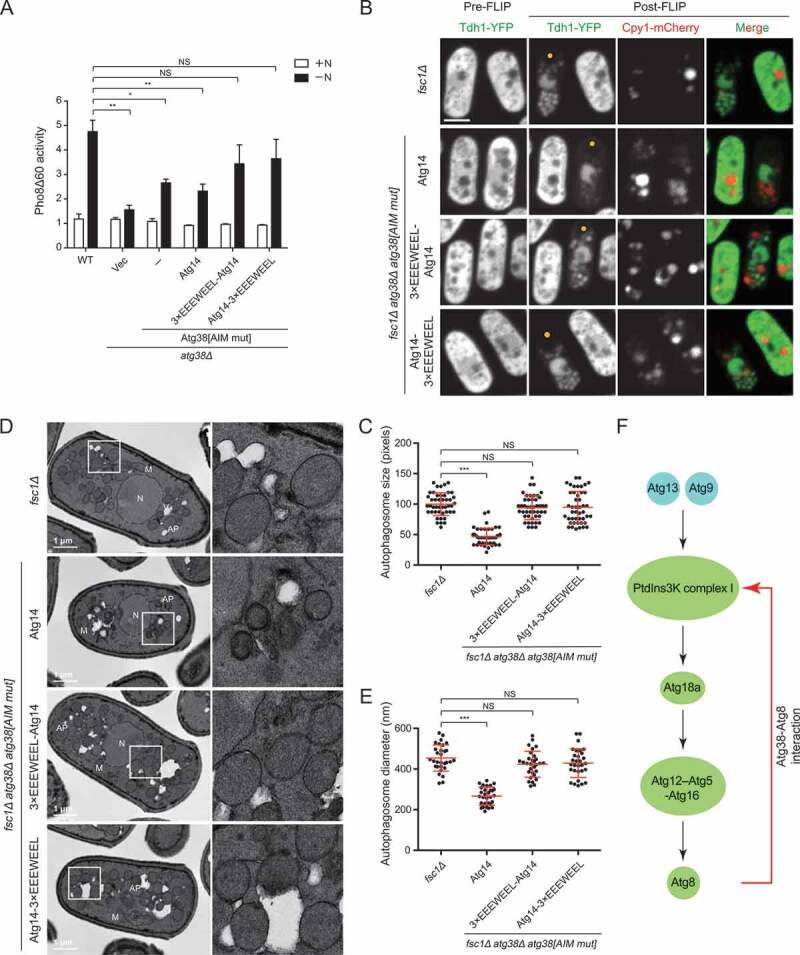
Artificially fusing an AIM to Atg14 rescues the autophagy defects caused by the Atg38 AIM mutation. (A) Autophagic flux measurement using the Pho8Δ60 assay was performed in wild-type cells, *atg38Δ* cells transformed with an empty vector or a plasmid expressing Atg38[AIM mut], and Atg38[AIM mut]-expressing *atg38Δ* cells transformed with a plasmid expressing Atg14 or a plasmid expressing Atg14 fused with an exogenous AIM at the N terminus or the C terminus. Cells were collected before (+N) and after shifting to nitrogen-free medium for 4 h (−N). Average activity from non-starved samples was set to 1. Data are mean ± s.d. of triplicates from representative experiments. * indicates *P* < 0.05; ** indicates *P* < 0.01; NS, not significant. *P* values were calculated using Welch’s t-test. (B) Representative images from the FLIP assay of *fsc1Δ* cells and *fsc1Δ atg38Δ* cells carrying Atg38[AIM mut] and the indicated Atg14 constructs. Cells expressing Tdh1-YFP were collected after 3 h of starvation, and then the FLIP assay was performed. Yellow dots mark the sites of photobleaching. Scale bar: 3 μm. (C) Quantification of the size of autophagosomes in (B). Mean ± s.d. are shown in red (n = 45). *** indicates *P* < 0.001; NS, not significant. *P* values were calculated using Welch’s t-test. (D) Representative electron microscopy images of *fsc1Δ* cells and *fsc1Δ atg38Δ* cells carrying Atg38[AIM mut] and the indicated Atg14 constructs. Cells were collected after 3 h of starvation. N, nucleus; M, mitochondrion; V, vacuole; AP, autophagosome. White squares enclosed regions are shown at higher magnification on the right. (E) Quantification of the diameter of autophagosomes in (D). Mean ± s.d. are shown in red (n = 30). *** indicates *P* < 0.001; NS, not significant. *P* values were calculated using Welch’s t-test. (F) The Atg38-Atg8 interaction establishes a positive feedback loop between Atg8 and the PtdIns3K complex I. The within-loop relationships denoted by black arrows are based on our previously published results [40]

If the main function of the Atg38-Atg8 interaction is to create a feedback loop between Atg8 and the PtdIns3K complex I, theoretically, it may be possible to substitute the Atg38-Atg8 interaction with an artificial interaction between Atg8 and another component of the PtdIns3K complex I. To test this idea, we fused an exogenous AIM (3×EEEWEEL) to the N terminus or the C terminus of Atg14 and expressed these chimeras in *atg38Δ* cells expressing Atg38[AIM mut]. Both the Pho8Δ60 assay and the Tdh1-YFP processing assay show that the autophagy defect of *atg38Δ* cells expressing Atg38[AIM mut] can be fully rescued by expressing 3×EEEWEEL-Atg14 or Atg14-3×EEEWEEL (Figure 7A **and S7A**). To exclude the possibility that this rescue is caused by the overexpression of Atg14, we expressed Atg14 in the same mutant and found that the autophagy defect remained unchanged. On the other hand, expressing 3×EEEWEEL-Atg14 or Atg14-3×EEEWEEL in wild-type cells did not elevate autophagy activity (**Fig. S7B**), suggesting that the increase of autophagy activity by 3×EEEWEEL-Atg14 or Atg14-3×EEEWEEL is specific to the Atg38 AIM mutation rather than a general effect. Consistent with the results from autophagic flux assays, the reduction of autophagosome size caused by the Atg38 AIM mutation was rescued by expressing 3×EEEWEEL-Atg14 or Atg14-3×EEEWEEL, but not Atg14 (Figure 7B**–**E).

Furthermore, PAS accumulation of Atg2, Atg18b, Atg24b, Atg5, Atg16, and Atg8 reduced by the Atg38 AIM mutation was recovered by expressing 3×EEEWEEL-Atg14 or Atg14-3×EEEWEEL (**Fig. S7C**). Together, these results show that an artificial interaction between Atg8 and the PtdIns3K complex I can obviate the need for the Atg38-Atg8 interaction, suggesting flexibility of how to establish a positive feedback loop between Atg8 and the PtdIns3K complex I.

## Discussion

In this study, we discovered that a fifth subunit of the PtdIns3K complex I in fission yeast, Atg38, interacts with Atg8 via an AIM. Through the Atg38-Atg8 interaction, Atg8 functions as a scaffold to recruit Atg38 to the PAS, thus establishing a positive feedback loop between Atg8 and the PtdIns3K complex I (Figure 7F). The positive feedback loop enhances PAS accumulation of the PtdIns3K complex I and of Atg proteins downstream of the PtdIns3K complex I and thereby promotes efficient phagophore expansion and autophagosome formation.

In budding yeast and mammals, Atg38 and NRBF2 have been reported to be a fifth subunit of the autophagy-specific PtdIns3K complex I [33–39]. Here we found that in fission yeast, besides Vps34, Vps15, Atg6, and Atg14, Atg38 is an additional subunit of the PtdIns3K complex I, confirming that the composition of the PtdIns3K complex I is conserved from yeasts to mammals. Interestingly, comparing with the observations that deletion of *atg38* in budding yeast decreases autophagy activity to about 50% [33], and knockout or knockdown of *NRBF2* in mammalian cells displays partially impaired [34–36] or enhanced [39] autophagy, fission yeast Atg38 appears more important for autophagy as deletion of *atg38* in fission yeast totally blocked autophagy activity and autophagosome formation (Figures 1B, 2A,B, and 4A,C). One possible explanation of the differing importance of Atg38/NRBF2 proteins in different species is that a functional AIM is present in fission yeast Atg38, but not in budding yeast Atg38 and mammalian NRBF2. Consistent with this view, we found that in fission yeast cells expressing Atg14 fused with an exogenous AIM, deletion of *atg38* only partially impaired autophagy (**Fig. S7B**), indicating a diminishment of the importance of Atg38 in this situation.

Although the AIM in fission yeast Atg38 is not conserved in non-fission-yeast species, AIM-mediated connections between Atg8 and the PtdIns3K complex I may still occur in these species through interactions between Atg8 and other subunit(s) of the PtdIns3K complex I. For example, mammalian ATG14 has recently been reported to interact with Atg8-family proteins GABARAP and GABARAPL1 via an AIM located within its BATS domain [30]. Our results that an artificial Atg14-Atg8 interaction can substitute the Atg38-Atg8 interaction also support flexibility of how Atg8 can be connected to the PtdIns3K complex I (Figure 7).

Previously, it has been shown that Atg38/NRBF2 contributes to the organization of the PtdIns3K complex I by linking the Vps15-Vps34 and Atg14-Atg6 subcomplexes and/or driving the dimerization of the PtdIns3K complex I [33,35,37–39]. In our study, autophagy defects are stronger in *atg38Δ* cells than in *atg38Δ* cells expressing Atg38[AIM mut] (Figure 2A,B**, and S4B**), indicating that Atg38 has role(s) beyond AIM-mediated binding with Atg8, probably in organizing the PtdIns3K complex I. Future biochemical and structural studies will be needed to confirm whether fission yeast Atg38 indeed has such a role and to reveal how this subunit is integrated into the PtdIns3K complex I.

AIMs were initially identified to be utilized in selective autophagy receptors for interacting with Atg8-family proteins, and later they were also found in core Atg proteins [17–30,54]. The identification of the Atg38 AIM in this study expands the list of AIMs in core Atg proteins, underscoring the ubiquity of the interactions between Atg8 and other core Atg proteins in autophagy. Two types of functional consequences have been proposed for the AIM-mediated interactions between Atg8 (GABARAP and LC3 proteins in mammals) and other core Atg proteins: (1) enhancing the activity of an upstream Atg protein, as exemplified by the ability of mammalian GABARAP to promote ULK1 kinase activation [27]; and (2) acting as a scaffold for the assembly of the upstream Atg proteins at the PAS or the phagophore, such as the roles of budding yeast Atg8 in bolstering the accumulation of Atg1, Atg3, and Atg4 at the PAS or the phagophore [21,23,25,55], and the roles of mammalian GABARAP proteins in promoting the localization of the ULK1 complex and the PtdIns3K complex I at autophagosomes [21,26,30]. Presumably, in both situations, these AIM-Atg8 interactions establish positive feedback loops between Atg8 and upstream AIM-containing proteins, and thereby endow special properties to the autophagosome biogenesis process.

Positive feedback loops are common in cellular signaling systems and can be established at the level of transcriptional regulation, post-transcriptional regulation, and post-translational regulation [56–59]. Positive feedback loops are expected to lead to bistability and hysteresis [60–62]. These properties may be the reason why AIM-mediated positive feedback loops are so ubiquitous in the autophagy machinery. Positive feedback loops can make autophagy induction a bi-stable process. The establishment of 2 discrete and stable steady states of autophagy non-induction and autophagy induction may allow cells to better respond to unpredictable and fluctuating environments. Further exploring the existence and mechanisms of positive feedback loops in autophagy in various organisms will help us gain a deeper understanding of autophagy.

## Materials and methods

### Fission yeast strains and plasmids

Fission yeast strains used in this study are listed in **Table S1**, and plasmids used in this study are listed in **Table S2**. Genetic methods for strain construction and composition of media are as described previously [63]. Most of the deletion strains used in this study were constructed by PCR amplifying the deletion cassettes in the Bioneer deletion strains and transforming the PCR products into our lab strains. The exception is *atg38*, whose deletion strains were made without the aid of Bioneer strains, by standard PCR-based gene targeting [64]. Strains expressing proteins fused with the YFP-FLAG-His_6_ (YFH) tag under native promoters were constructed by an overlap-extension PCR approach [65], using the Yoshida ORFeome plasmids as template [66]. Strains expressing CFP-Atg8 and Tdh1-YFH were constructed as reported previously [40]. Strains expressing Atg17-mCherry under the native promoter were constructed by PCR-based tagging. Plasmids expressing proteins under the control of the *nmt1* or *41nmt1* (medium-strength *nmt1* promoter) promoter were constructed using vectors modified from pDUAL-YFH1c (REKIN BioResource Research Center, RDB06151) [67]. Plasmids expressing 13MYC-Atg38, 13MYC-Atg38[AIM mut], or 13MYC-Atg38[AIM mut]-3×EEEWEEL were constructed by inserting the *atg38* promoter and sequences encoding Atg38, Atg38[AIM mut], or Atg38[AIM mut]-3×EEEWEEL into a modified pDUAL vector, which contains the sequence encoding 13MYC. Plasmids expressing mCherry-Atg14, mCherry-3×EEEWEEL-Atg14, or mCherry-Atg14-3×EEEWEEL were constructed by inserting the coding sequence of Atg14, 3×EEEWEEL-Atg14, or Atg14-3×EEEWEEL into modified pDUAL vectors, which contain the *41nmt1* promoter and the sequence encoding mCherry. Plasmids expressing CFP-Atg8, CFP-Atg8[AB mut], or CFP-Atg8[1–115] were constructed by inserting the *atg8* promoter and the coding sequence of Atg8, Atg8[AB mut], or Atg8[1–115] into a modified pDUAL vector, which contains the sequence encoding CFP. The above pDUAL plasmids were linearized with NotI digestion and integrated at the *leu1* locus or linearized with MluI digestion and integrated at the *ars1* replication origin region upstream of the *hus5* gene. Plasmids expressing Atg38-3×GFP or Atg38[AIM mut]-3×GFP were constructed by inserting the *atg38* promoter and the coding sequence of Atg38 or Atg38[AIM mut] into the vector pFA6a-3×GFP-kanMX6 (a gift from Dr. Jian-Qiu Wu, The Ohio State University, Columbus, USA) [68]. The resulting plasmids were linearized with AgeI digestion that cuts within the *atg38* promoter and integrated at the *atg38* locus. A pDUAL plasmid expressing GFP-tagged *S. cerevisiae* Pho8Δ60 under the *41nmt1* promoter was constructed by amplifying the DNA sequence encoding *S. cerevisiae* Pho8Δ60 from an *S. cerevisiae* Pho8Δ60 strain (a gift from Dr. Zhiping Xie, Shanghai Jiao Tong University, Shanghai, China) and cloning it into a pDUAL vector.

### PB *transposon-based genetic screen for autophagy genes in fission yeast*

In the screening, we first constructed an *isp6Δ* strain containing integrated *PB* and *nmt1* promoter-driven PBase (DY44358). This strain was pre-cultured in liquid nutrient-rich medium (EMM; 0.3% potassium hydrogen phthalate [Sigma-Aldrich, P1088], 0.5% NH_4_Cl [Sigma-Aldrich, A9434], 0.22% Na_2_HPO_4_ [Sinopharm, 20040618], 2% glucose [Sinopharm, 10010518], 0.105% MgCl_2_ · 6H_2_O [Sinopharm, 10012818], 0.00147% CaCl_2_ · 2H_2_O [Sigma-Aldrich, C7902], 0.1% KCl [Sigma-Aldrich, P9541], 0.004% Na_2_SO_4_ [Sigma-Aldrich, 239313], 0.0001% pantothenic acid [Sigma-Aldrich, P5155], 0.001% nicotinic acid [Sigma-Aldrich, N0761], 0.001% inositol [Sigma-Aldrich, I7508], 0.000001% biotin [Sigma-Aldrich, B4639], 0.00005% boric acid [Beijing Chemical Works, A0300001], 0.000045% MnSO_4_·H_2_O [Macklin, M813649], 0.00004% ZnSO_4_ · 7H_2_O [Sinopharm, 10024018], 0.00002% FeCl_3_ · 6H_2_O [Sigma-Aldrich, F2877], 0.000004% molybdic acid [Macklin, M813126], 0.00001% potassium iodide [Beijing Chemical Works, A1049007], 0.000004% CuSO_4_ · 5H_2_O [Sigma-Aldrich, 469130], 0.0001% citric acid·H_2_O [Beijing Chemical Works, B0803001]) with arginine (Sigma-Aldrich, A6969) and thiamine (Sinopharm, 67002134). After 5 to 6 generations growth, cells were washed twice with double-distilled water and transferred to EMM medium without thiamine for at least 40 h to allow the expression of PBase and induce the mutagenesis. Then the cells were spread on EMM plates containing thiamine, but not arginine, to select for Arg^+^ cells (approximately 0.1 OD_600_ unit of cells for each 9-cm plate). After 2 d at 30°C, EMM plates were replica-plated to nitrogen-free medium (EMM−N; EMM without NH_4_Cl) for 4 d of starvation. After starvation treatment, the colonies were replica-plated from EMM−N medium to YES (0.5% yeast extract [OXOID, LP0021], 3% glucose [Sinopharm, 10010518], 0.02% leucine [Sigma-Aldrich, L8912], 0.02% histidine [Sigma-Aldrich, H6034], 0.022% adenine sulfate dihydrate [Amresco, 0607], 0.02% uracil [Sigma-Aldrich, U1128]) plates, which were then incubated at 25°C. Colonies grown up most rapidly on YES plates were collected to identify the insertion sites of *PB* with inverse PCR or high-throughput sequencing as described previously [51,69].

### AP-MS analysis

AP-MS analysis was performed as described previously [43]. For each sample, approximately 1000–2000 OD_600_ units of cells were harvested after 2 h of starvation.

### Cpy1 (carboxypeptidase Y) colony blot assay

The Cpy1 colony blot assay was adapted from the corresponding assay in *S. cerevisiae* [70]. Fission yeast strains containing mCherry-tagged Cpy1 were transferred from 96-well plates to YES plates using a pinning tool and incubated at 30°C for 2 to 3 d. Once strains had grown into round patches, they were overlaid with a nitrocellulose membrane (GE Healthcare Life Sciences, 10600002). The plate was then incubated at 30°C for 24 h. The nitrocellulose membrane was washed 3 times with double-distilled water and then with standard phosphate-buffered saline. The membrane was then subjected to immunoblotting with an antibody against mCherry (Abmart, 4C16).

### CFP-Atg8 processing assay and Tdh1-YFP processing assay

For CFP-Atg8 processing assay, cell lysates were prepared from approximately 10 OD_600_ units of cells using a post-alkaline extraction method [71]. For Tdh1-YFP processing assay, cell lysates were prepared from approximately 3 OD_600_ units of cells using a trichloroacetic acid (TCA) (Sigma-Aldrich, T0699) lysis method [72]. Samples were separated by 12% SDS-PAGE and immunoblotted with an antibody against GFP (Roche, 11814460001), which also recognizes CFP and YFP. Protein bands were quantified using the “Analyze tool” in the Fiji distribution of the ImageJ software (National Institutes of Health) [73].

### Pho8∆60 assay in fission yeast

For the Pho8Δ60 assay, *S. pombe pho8Δ* strain transformed with a plasmid expressing GFP-tagged *S. cerevisiae* Pho8Δ60 under the *41nmt1* promoter was used as the assay strain background. Enzymatic activity was measured as described in *S. cerevisiae* [52] with some modifications. Cells with expected genotypes were cultured to mid-log phase in EMM medium before they were shifted to EMM−N medium for 4 h. Five OD_600_ units of cells were harvested at each time point and washed with 0.85% NaCl (Sinopharm, 10019318). Then the cells were suspended in 200 μl of lysis buffer (20 mM PIPES [Sigma-Aldrich, P6757], pH 6.8, 50 mM KCl [Sigma-Aldrich, P9541], 100 mM KOAc [Beijing Yili Fine Chemicals, 127-08-2], 10 mM MgSO_4_ [Sigma-Aldrich, 2643], 10 μM ZnSO_4_ [Sinopharm, 10024018], 0.5% Triton X-100 [Amersham Biosciences, 17-1315-01], 2 mM PMSF [Amresco, M145]) and incubated at room temperature for 20 min. PMSF was added to the final concentration of 4 mM and the samples were disrupted by vortexing with 0.5-mm-diameter glass beads immediately using a FastPrep-24 instrument. After centrifugation, 50 μl of the supernatant was added to 400 μl of reaction buffer (250 mM Tris∙HCl [Amresco, 497; Sinopharm, 10011018], pH 8.5, 10 mM MgSO_4_ [Sigma-Aldrich, 2643], 10 μM ZnSO_4_ [Sinopharm, 10024018], 0.4% Triton X-100 [Amersham Biosciences, 17-1315-01], 5.5 mM 1-naphthyl phosphate disodium salt [Sigma-Aldrich, N7255]) to start the reaction. After incubation at 30°C for 20 min, 500 μl of 1 M glycine-NaOH (pH 11.0; Amresco, 167; Sigma-Aldrich, S8045) was added to stop the reaction. Fluorescence intensity of emission at 472 nm after excitation at 345 nm was measured. Protein concentration was determined by the BCA method (Thermo, 23227).

### Western blotting of fluorescent-protein-fused or MYC-fused proteins

Cell lysates were prepared from approximately 3 OD_600_ units of cells using a trichloroacetic acid (TCA) lysis method [72]. Samples were separated by 8% or 10% SDS-PAGE and then immunoblotted with an antibody against GFP/CFP/YFP (Roche, 11814460001) or MYC (Abmart, M20002L).

### Atg8 lipidation assay

Cells were collected after shifting to nitrogen-free medium (−N) for 8 h. During nitrogen starvation, 1 mM PMSF (Amresco, M145) was added to the culture at the time point of −N 0, 2, 4, and 6 h. Cell lysates were prepared from approximately 3 OD_600_ units of cells using a trichloroacetic acid (TCA) lysis method [72]. Samples were separated by 15% SDS-PAGE containing 6 M urea (Sigma-Aldrich, U0631) and then immunoblotted with an antibody against CFP (Roche, 11814460001).

### Immunoprecipitation

Approximately 100 OD_600_ units of cells were harvested and washed once with double-distilled water. Cells were lysed by beating with 0.5-mm-diameter glass beads in the lysis buffer (50 mM HEPES [Amresco, 511], pH 7.5, 150 mM NaCl [Sinopharm, 10019318], 1 mM EDTA [Sigma-Aldrich, E5134], 1 mM DTT [Amresco, 281], 1 mM PMSF [Amresco, M145], 0.05% NP-40 [Sigma-Aldrich, 74385], 10% glycerol [Beijing Chemical Works, B0304001], 1× Roche protease inhibitor cocktail [Roche, 4693132001]) using a FastPrep-24 instrument. The supernatants of cell lysates were incubated with mCherry-Trap agarose beads. After incubation, the agarose beads were washed 3 times with lysis buffer. Proteins bound to beads were eluted by boiling in SDS-PAGE loading buffer.

### Yeast two-hybrid assay

For yeast two-hybrid assay, the MATCHMAKER Two-Hybrid System 3 (CLONTECH Laboratories, K1612-1) was used. Bait plasmids and prey plasmids were constructed by inserting cDNAs into a modified pGBKT7 (CLONTECH Laboratories, K1612-B) or pGADT7 (CLONTECH Laboratories, K1612-A) vector, respectively. Bait and prey plasmids were co-transformed into the strain AH109 (CLONTECH Laboratories, K1612-1), and transformants were selected on the double dropout medium (SD/−Leu/−Trp). The activation of the *HIS3* reporter gene was assessed on the triple dropout medium (SD/−His/−Leu/−Trp), and the activation of the *HIS3* and *ADE2* reporter genes was assessed on quadruple dropout medium (SD/−Ade/−His/−Leu/−Trp).

### GST affinity-isolation assay

GST, GST-Atg38[161–190], GST-Atg38[161–190]^F178A,V181A^, HA-Atg8, and HA-Atg8^P52A,R67A^ were expressed in the BL21 (DE3) *E. coli* strain (Sigma-Aldrich, CMC0014), respectively. *E. coli* cells were lysed in lysis buffer (1× PBS [0.8% NaCl (Sinopharm, 10019318), 0.02% KCl (Sigma-Aldrich, P9541), 0.144% Na_2_HPO_4_ (Sinopharm, 20040618), 0.024% KH_2_PO_4_ (Beijing Yili Fine Chemicals, 7778-77-0)], pH 7.5, 200 mM NaCl [Sinopharm, 10019318], 10% glycerol [Beijing Chemical Works, B0304001], 0.05% NP-40 [Sigma-Aldrich, 74385], 1 mM PMSF [Amresco, M145]) by sonication. The supernatants of cell lysates containing GST alone or GST-fused Atg38 constructs were mixed with the supernatants of cell lysates containing HA-fused Atg8 constructs and incubated at 4°C for 0.5 h before bound to glutathione Sepharose. Then the samples were incubated at 4°C for 2 h. Beads were washed 3 times with lysis buffer without PMSF and subsequently eluted by SDS-PAGE loading buffer. The supernatants of cell lysates and incubated beads were analyzed by SDS-PAGE and stained with Coomassie Brilliant Blue R-250 (Amresco, 0472) or immunoblotted with an antibody against HA (MBL, M180-3).

### Fluorescence microscopy

Except for the FLIP assay, live-cell imaging was performed using a DeltaVision PersonalDV system (Applied Precision) equipped with an mCherry/YFP/CFP filter set (Chroma 89006 set) and a Photometrics CoolSNAP HQ2 camera. Images were acquired with a 100×, 1.4-NA objective and analyzed with the SoftWoRx software (GE Healthcare Life Sciences). Puncta intensity was determined by measuring the fluorescence intensities of 2 concentric square regions (5 × 5 and 7 × 7 pixels, respectively) centered around the punctum and by performing a background subtraction calculation [74].

### FLIP assay

The photobleaching of the Tdh1-YFP signal and image acquisition were performed with a PerkinElmer Ultraview VoX spinning-disc system, using a 100× objective. Quantitative analysis of autophagosome size was performed with the Volocity software (Quorum Technologies).

### Electron microscopy

Approximately 30 OD_600_ units of cells were starved for 3 h and harvested, and then washed once with 10 ml of EMM−N medium. The samples were fixed with 1% glutaraldehyde (Electron Microscopy China, GS2607) and 4% KMnO_4_ (Sinopharm, 10017318), and then dehydrated through a graded ethanol series and embedded in Spurr’s resin (Electron Microscopy Sciences, 14300). Thin sections were examined using a FEI Tecnai G2 Spirit electron microscope equipped with a Gatan 895 4k×4k CCD camera. The sizes of autophagosomes were determined as described previously [41].

### Statistical analysis

Data are presented as mean ± s.d. A two-tailed, unpaired Welch’s t-test was used for calculation of *P* values. *P* values of < 0.05 were considered significant.

## Supplementary Material

Supplemental Material
