# Supplementary material for: Atg38-Atg8 interaction in fission yeast establishes a positive feedback loop to promote autophagy
Source: Autophagy. 2020 Jan 19;16(11):2036–51. doi: 10.1080/15548627.2020.1713644 (PMC7595586; doi:10.1080/15548627.2020.1713644)
Supplement: Supplemental Material [file KAUP_A_1713644_SM4491.doc]

**Supplementary Information**

**Atg38-Atg8 interaction in fission yeast establishes a positive feedback loop to promote autophagy**

Zhong-Qiu Yu, Ling-Ling Sun, Zhao-Di Jiang, Xiao-Man Liu, Dan Zhao, Hai-Tao Wang, Wan-Zhong He, Meng-Qiu Dong, Li-Lin Du

TABLE OF CONTENTS

Figure S1 ................................................................................................... 2

Figure S2 ................................................................................................... 4

Figure S3 ................................................................................................... 5

Figure S4 ................................................................................................... 7

Figure S5 ................................................................................................... 9

Figure S6 .................................................................................................. 10

Figure S7 .................................................................................................. 11

Table S1 ................................................................................................. 13

Table S2 ................................................................................................. 22


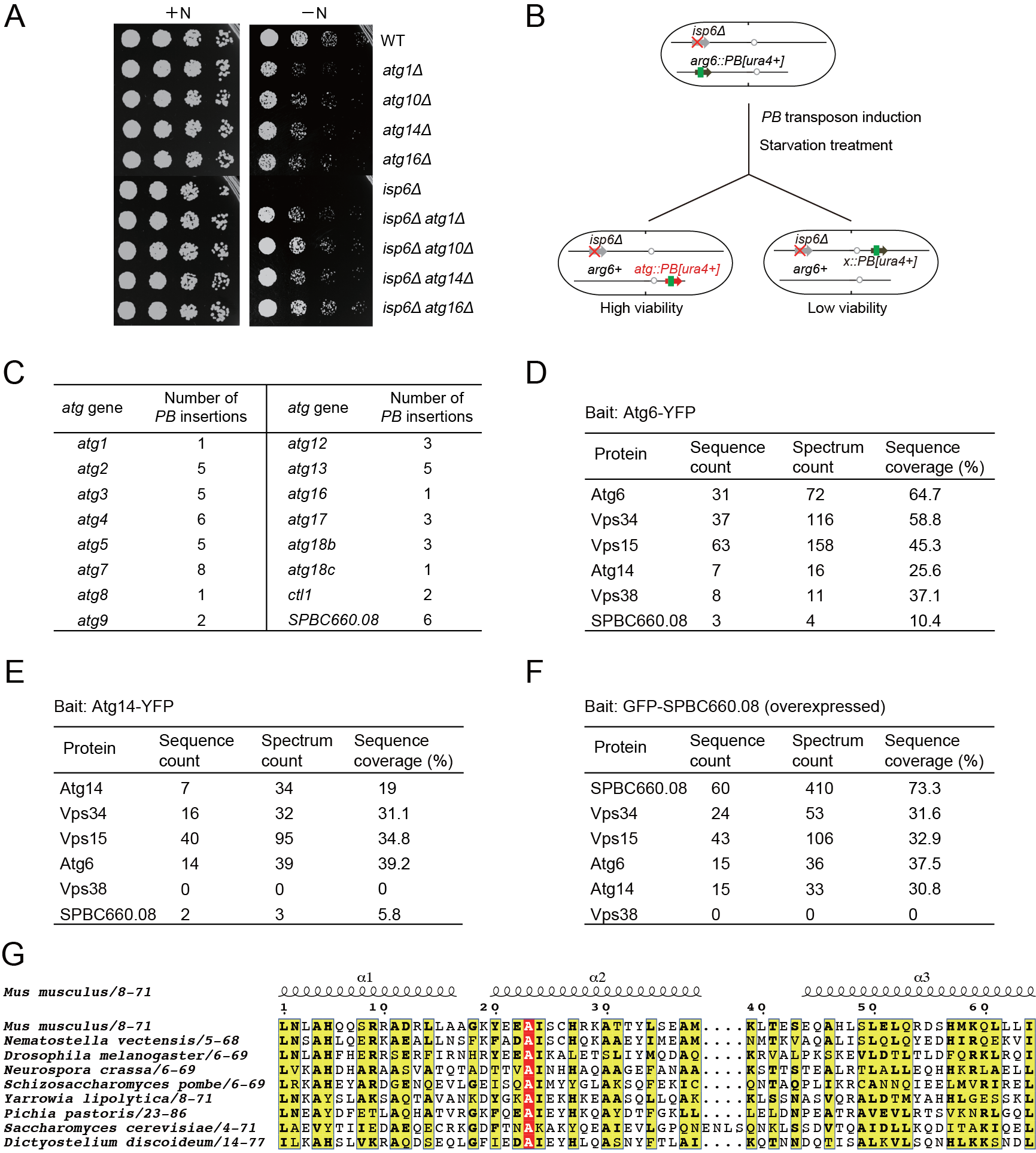


**Figure S1.** SPBC660.08/Atg38 is a subunit of the PtdIns3K complex I in fission yeast. (**A**) Suppression of nitrogen-starvation-induced viability loss of *isp6Δ* by deleing *atg1*, *atg10*, *atg14*, or *atg16*. Mid-log phase cells grown in nutrient-rich medium were either untreated (+N), or transferred to liquid nitrogen-free medium and incubated at 30°C for 4 d (−N). Aliquots of cells from each culture were spotted onto YES medium in sequential 5-fold dilutions. (**B**) Schematic of *piggyBac* (*PB*) transposon-based genetic screen for autophagy genes. (**C**) Summary of the autophagy factors identified in the *isp6Δ* suppressor screen using *PB* transposon-based mutagenesis. (**D-F**) Mass spectrometry data of PtdIns3K complexes subunits from the results of affinity purification coupled with mass spectrometry (AP-MS) analysis using Atg6-YFP, Atg14-YFP, or GFP-SPBC660.08 as bait. Cells expressing Atg6-YFP, Atg14-YFP, or GFP-SPBC660.08 were collected after 2 h of starvation, and AP-MS analysis was performed. (**G**) Multiple sequence alignment of the MIT domains in Atg38/NRBF2-family proteins. The alignment was generated with Jalview. Secondary structural elements of the MIT domain of mouse NRBF2 (PDB 2crb) were visualized together with the sequence alignment using the ESPript 3.0 web server (http://espript.ibcp.fr/). Protein sequence accession numbers are gi|81879255 (*Mus musculus*), gi|156407013 (*Nematostella vectensis*), gi|281365402 (*Drosophila melanogaster*), gi|85108754 (*Neurospora crassa*), gi|429239516 (*Schizosaccharomyces pombe*), gi|50550267 (*Yarrowia lipolytica*), gi|254565691 (*Pichia pastoris*), gi|84027749 (*Saccharomyces cerevisiae*), and gi|66806327 (*Dictyostelium discoideum*). Identical and similar residues are boxed in red and yellow, respectively.


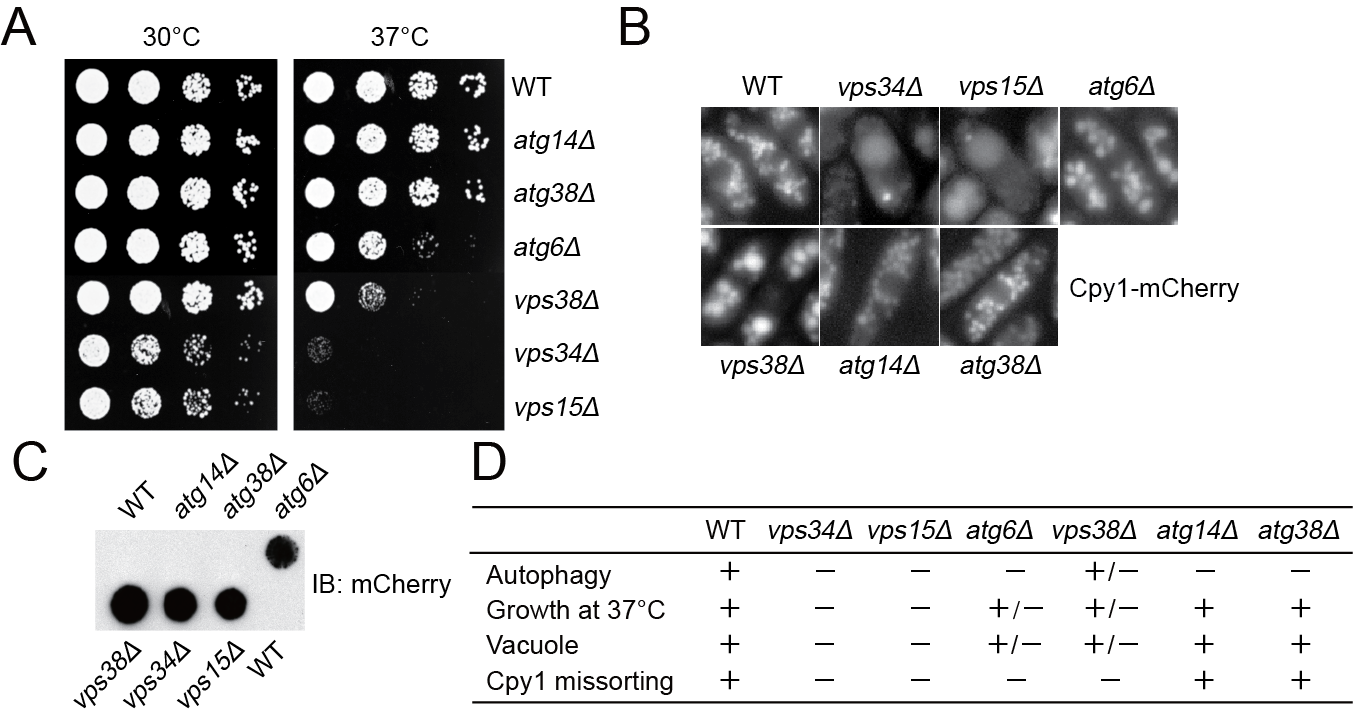


**Figure S2.** Deletion of *atg38* does not exhibit phenotypes characteristic of the loss of functional PtdIns3K complex II. (**A**) Growth properties of mutants lacking individual subunits of PtdIns3K complexes. Deleting *vps34* is known to cause sensitivity to high temperature and other stresses [1,2]. (**B**) Vacuole morphology of mutants lacking individual subunits of PtdIns3K complexes under normal vegetative growth conditions. Cells expressing Cpy1-mCherry were imaged by fluorescence microscopy. Deleting *vps34* is known to cause vacuole enlargement [1,2]. (**C**) The colony blot assay was used to determine the extent of missorting of Cpy1-mCherry to cell surface. Deleting *vps34* is known to cause Cpy1 missorting [3]. (**D**) Summary of the phenotypes of removing individual subunits of PtdIns3K complexes.


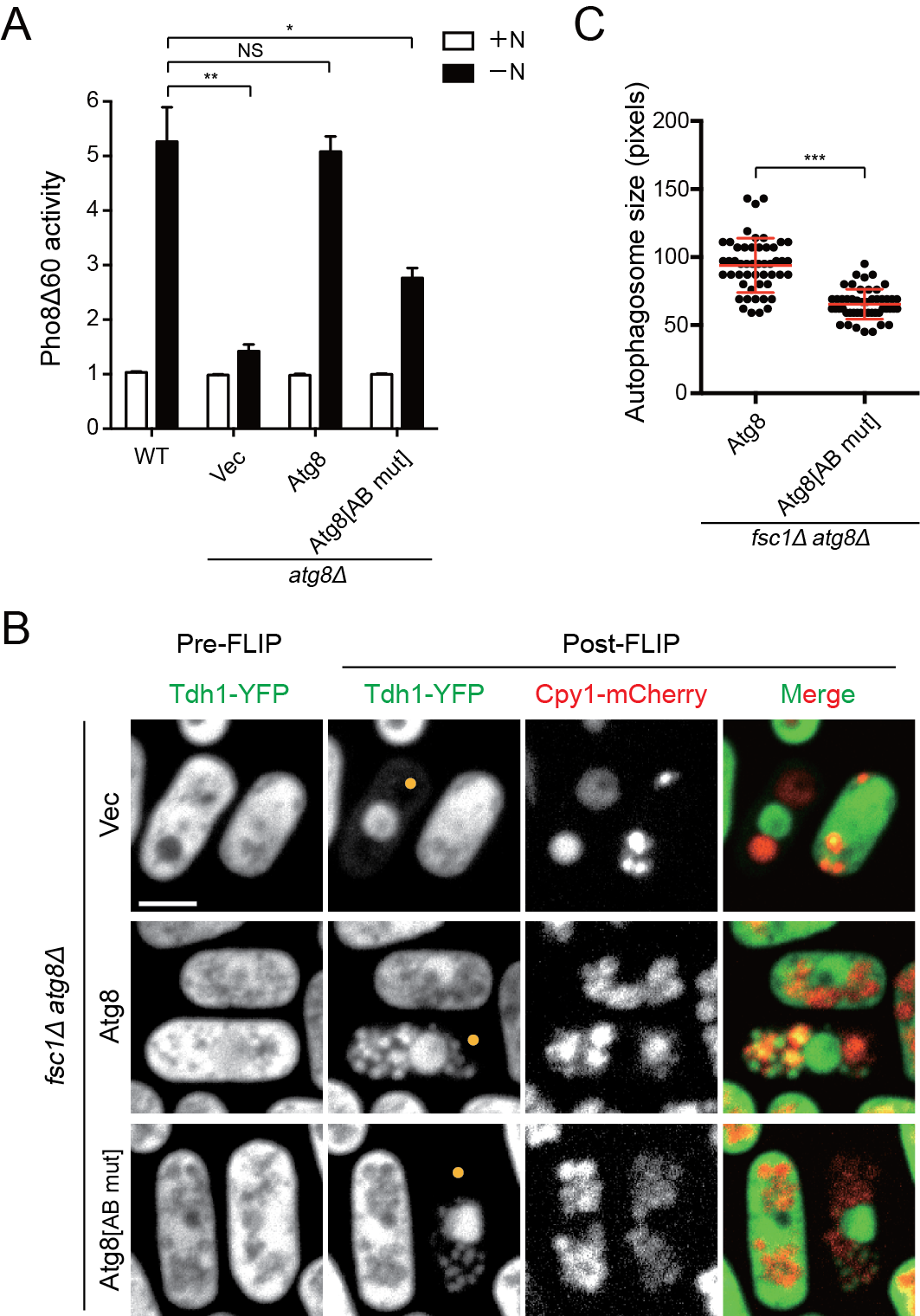


**Figure S3.** The AIM-binding region mutation in Atg8 impairs autophagy. (**A**) Autophagic flux measurement using the Pho8Δ60 assay was performed in wild-type cells and *atg8Δ* cells transformed with an empty vector or a plasmid expressing wild-type Atg8 or Atg8[AB mut]. Cells were collected before (+N) and after shifting to nitrogen-free medium for 4 h (−N). Average activity from non-starved samples was set to 1. Data are mean ± s.d. of triplicates from representative experiments. * indicates *P* < 0.05; ** indicates *P* < 0.01; NS, not significant. *P* values were calculated using Welch’s t-test. (**B**) Representative images from the FLIP assay of *fsc1Δ atg38Δ* cells transformed with an empty vector or a plasmid expressing wild-type Atg8 or Atg8[AB mut]. The FLIP assay was performed to examine autophagosome formation in Atg8[AB mut] cells. Cells expressing Tdh1-YFP were collected after 3 h of starvation, and then the FLIP assay was performed. Yellow dots mark the sites of photobleaching. Scale bar: 3 μm. (**C**) Quantification of the size of autophagosomes in (B). Mean ± s.d. are shown in red (n = 45). *** indicates *P* < 0.001. *P* values were calculated using Welch’s t-test.


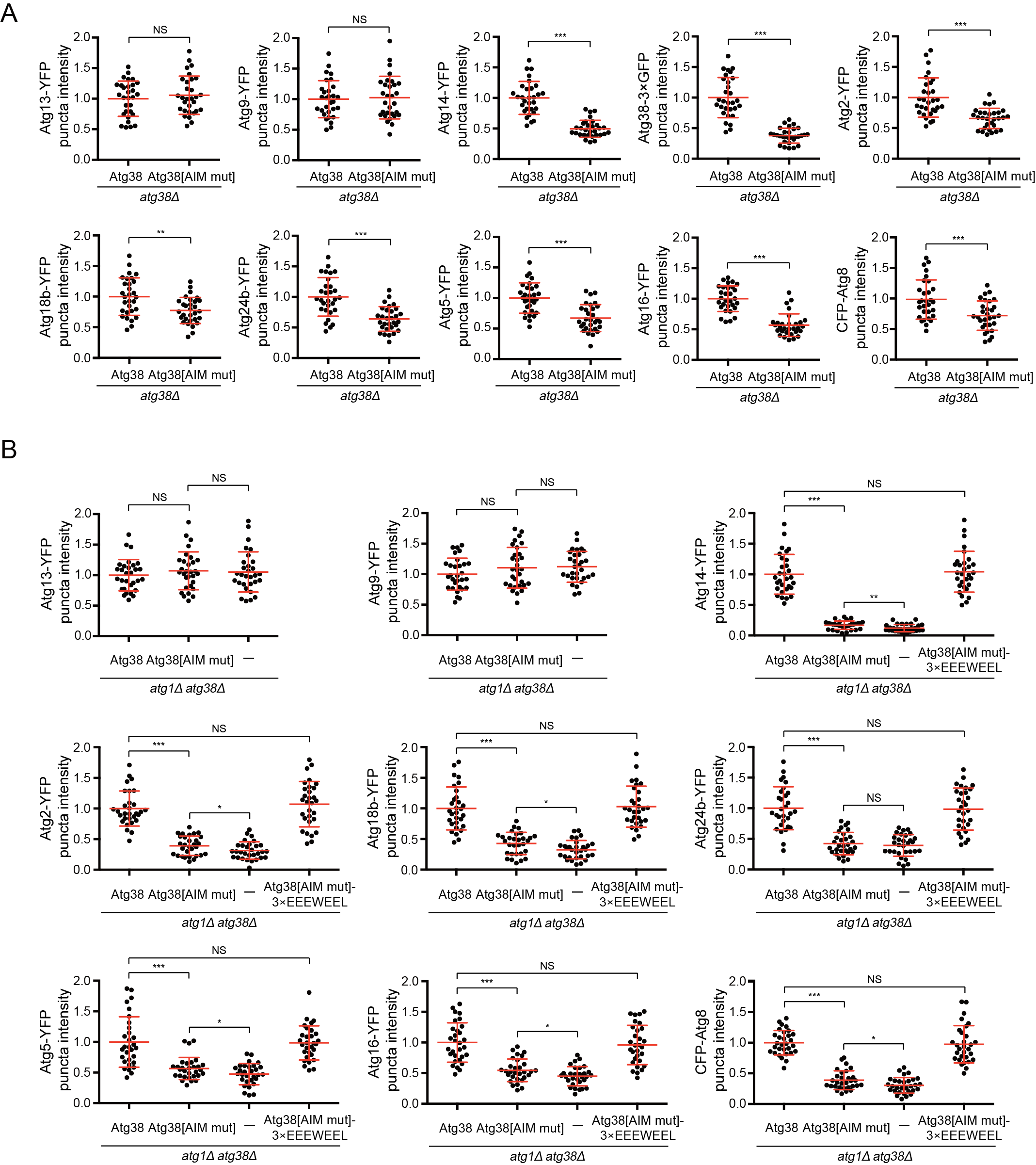


**Figure S4.** The influence of the Atg38 AIM mutation on PAS localization of Atg proteins. (**A**) In the wild-type background, the AIM mutation in Atg38 influences PAS accumulation of the PtdIns3K complex I and of Atg proteins downstream of the PtdIns3K complex I, but not of Atg proteins upstream of the PtdIns3K complex I. Quantification of the puncta intensity of Atg proteins at the PAS in *atg38Δ* cells expressing Atg38 or Atg38[AIM mut]. (**B**) In the *atg1Δ* background, inserting an exogenous AIM in Atg38[AIM mut] recovered the PAS accumulation of the Atg proteins reduced by the Atg38 AIM mutation. Quantification of the puncta intensity of Atg proteins at the PAS in *atg38Δ* cells and *atg38Δ* cells expressing Atg38, Atg38[AIM mut], or Atg38[AIM mut] inserted with an exogenous AIM (3×EEEWEEL) between Ala181 and Glu182. Mid-log phase cells expressing YFP, CFP, or 3×GFP-tagged Atg proteins were incubated in nitrogen-free medium for 2 h, and then imaged by fluorescence microscopy. Mean ± s.d. are shown in red (n = 30). * indicates *P* < 0.05; ** indicates *P* < 0.01; *** indicates *P* < 0.001; NS, not significant. *P* values were calculated using Welch’s t-test.


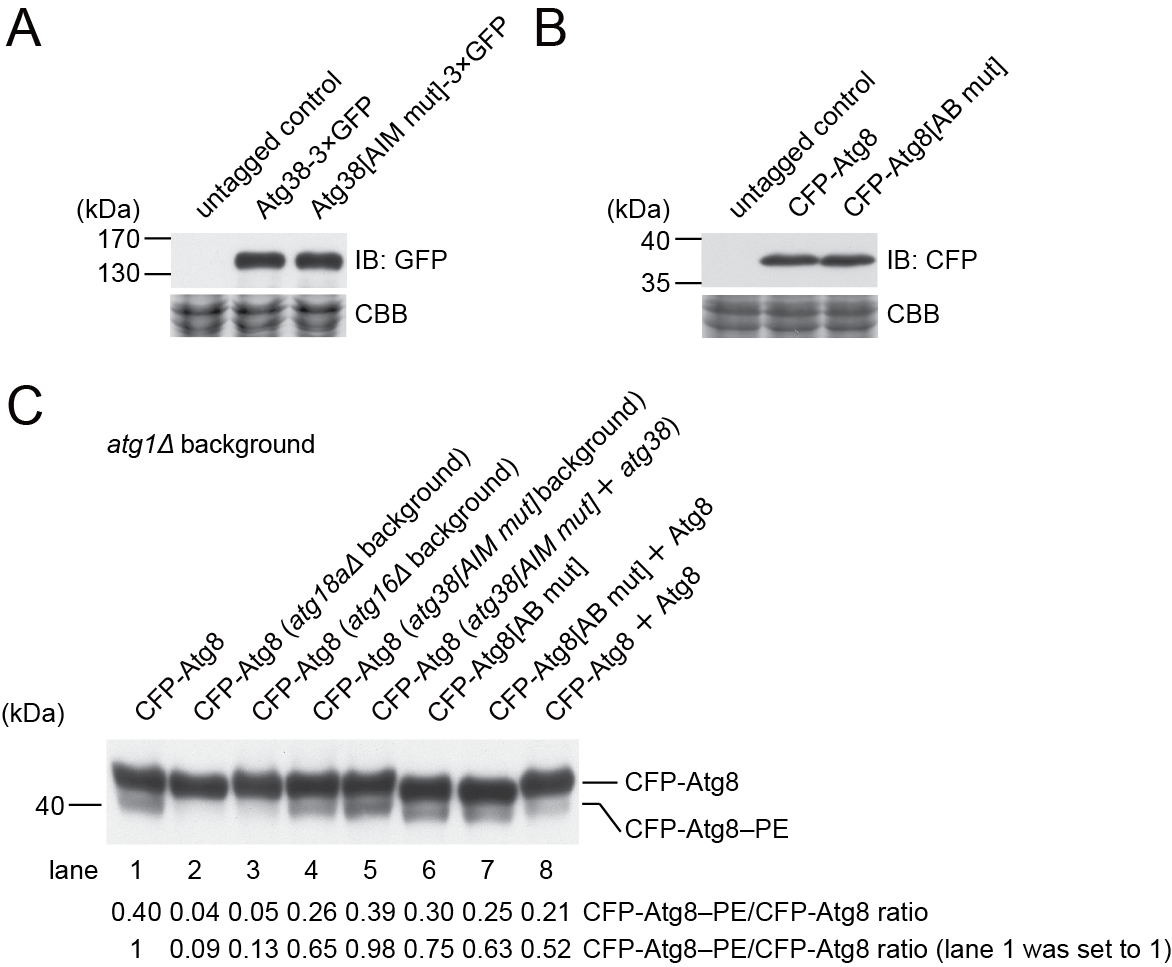


**Figure** **S5.** The Atg38-Atg8 interaction does not directly enhance the lipidation of Atg8. (**A**) The protein levels of wild-type Atg38 and AIM-mutated Atg38. Cells expressing 3×GFP-tagged Atg38 or Atg38[AIM mut] and an untagged control were collected after 2 h of starvation, and then analyzed by immunoblotting with antibody against GFP. (**B**) The protein levels of wild-type Atg8 and AIM-binding defective Atg8. Cells expressing CFP-tagged Atg8 or Atg8[AB mut] and an untagged control were collected after 2 h of starvation, and then analyzed by immunoblotting with antibody against CFP. Coomassie Brilliant Blue R-250 (CBB) staining of PVDF membrane after immunodetection served as protein loading control. (**C**) The lipidation status of Atg8 in different mutants. Cells expressing CFP-tagged Atg8 or Atg8[AB mut] were collected after 8 h of starvation, and then analyzed by immunoblotting with antibody against CFP.


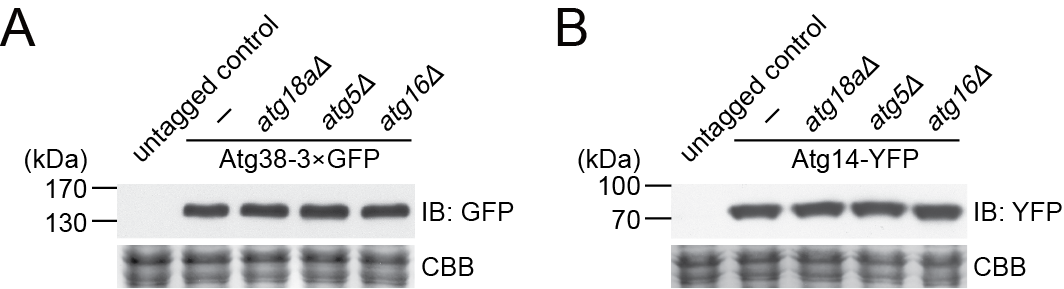


**Figure S6.** The loss of Atg18a, Atg5, or Atg16 does not affect the protein levels of Atg38 and Atg14. (**A**) The protein level of Atg38 in *atg18aΔ*, *atg5Δ*, or *atg16Δ* cells. Cells expressing 3×GFP-tagged Atg38 and an untagged control were collected after 2 h of starvation, and then analyzed by immunoblotting with antibody against GFP. (**B**) The protein level of Atg14 in *atg18aΔ*, *atg5Δ*, or *atg16Δ* cells. Cells expressing YFP-tagged Atg14 and an untagged control were collected after 2 h of starvation, and then analyzed by immunoblotting with antibody against YFP. Coomassie Brilliant Blue R-250 (CBB) staining of PVDF membrane after immunodetection served as protein loading control.


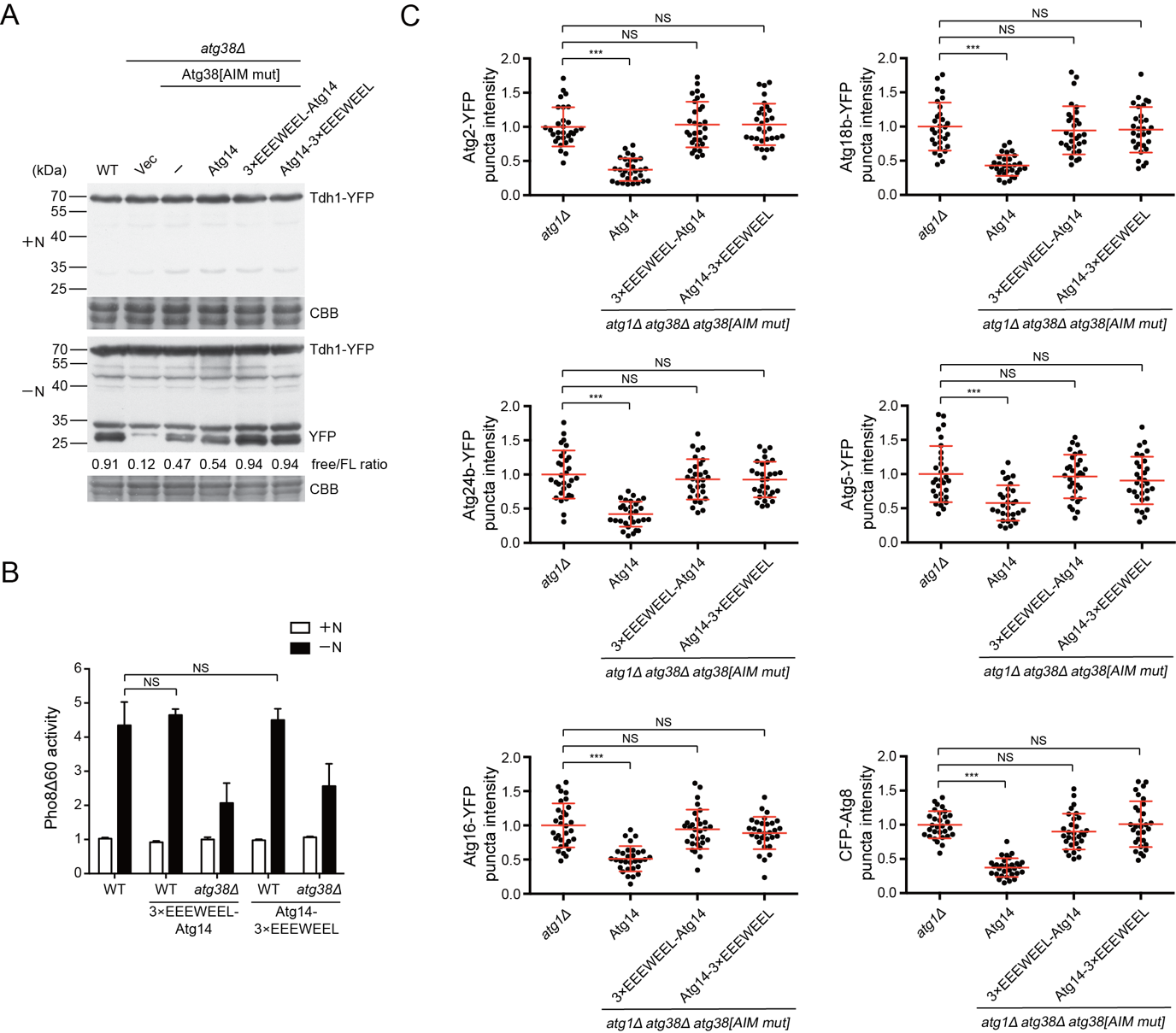


**Figure S7.** Artificially fusing an AIM to Atg14 rescues the autophagic flux defect and reduced PAS accumulation of Atg proteins caused by the Atg38 AIM mutation. (**A**) Starvation-induced processing of Tdh1-YFP was examined in wild-type cells, *atg38Δ* cells transformed with an empty vector or a plasmid expressing Atg38[AIM mut], and Atg38[AIM mut]-expressing *atg38Δ* cells transformed with a plasmid expressing Atg14 or a plasmid expressing Atg14 fused with an exogenous AIM at the N terminus or the C terminus. Cells expressing YFP-tagged Tdh1 were collected before (+N) and after shifting to nitrogen-free medium for 2 h (−N), and the total lysates were analyzed by immunoblotting with antibody against YFP. Coomassie Brilliant Blue R-250 (CBB) staining of PVDF membrane after immunodetection served as protein loading control. (**B**) Autophagic flux measurement using the Pho8Δ60 assay was performed in wild-type or *atg38Δ* cells expressing Atg14 fused with an exogenous AIM at the N terminus or the C terminus. Cells were collected before (+N) and after shifting to nitrogen-free medium for 4 h (−N). Average activity from non-starved samples was set to 1. Data are mean ± s.d. of triplicates from representative experiments. NS, not significant. *P* values were calculated using Welch’s t-test. (**C**) Quantification of the puncta intensity of Atg proteins at the PAS in *atg38Δ* cells expressing Atg38, and Atg38[AIM mut]-expressing *atg38Δ* cells transformed with a plasmid expressing Atg14 or a plasmid expressing Atg14 fused with an exogenous AIM at the N terminus or the C terminus. Mid-log phase cells expressing YFP, or CFP-tagged Atg proteins were incubated in nitrogen-free medium for 2 h, and then imaged by fluorescence microscopy. Mean ± s.d. are shown in red (n = 30). *** indicates *P* < 0.001; NS, not significant. *P* values were calculated using Welch’s t-test. Note that quantification data of *atg38Δ* cells expressing Atg38 are from the same experiments of those shown in Figure S4B.

**Reference**

1. Kimura K, Miyake S, Makuuchi M, Morita R, Usui T, Yoshida M, et al. Phosphatidylinositol-3 Kinase in Fission Yeast: A Possible Role in Stress Responses. Biosci Biotechnol Biochem. 1995;59: 678–682. doi:10.1271/bbb.59.678

2. Takegawa K, DeWald DB, Emr SD. Schizosaccharomyces pombe Vps34p, a phosphatidylinositol-specific PI 3-kinase essential for normal cell growth and vacuole morphology. J Cell Sci. 1995;108: 3745–3756.

3. Cheng H. Role of the Rab GTP-Binding Protein Ypt3 in the Fission Yeast Exocytic Pathway and Its Connection to Calcineurin Function. Mol Biol Cell. 2002;13: 2963–2976. doi:10.1091/mbc.01-09-0463

**Table S1.** Strains used in this study

| Strain | Mating Type | Genotype | Use |
| --- | --- | --- | --- |
| DY3510 | h− | *leu1-32 CFP-atg8::leu+* | Figure 1B and S1A |
| DY44347 | h+ | *leu1-32 vps38Δ::kanMX CFP-atg8::leu1+* | Figure 1B |
| DY11898 | h? | *leu1-32 atg6Δ::kanMX CFP-atg8::leu1+* | Figure 1B |
| DY44348 | h+ | *leu1-32 vps15Δ::kanMX CFP-atg8::leu1+* | Figure 1B |
| DY44349 | h? | *leu1-32 vps34Δ::kanMX CFP-atg8::leu1+* | Figure 1B |
| DY11931 | h− | *leu1-32 atg14Δ::kanMX CFP-atg8::leu1+* | Figure 1B and S1A |
| DY44350 | h− | *leu1-32 atg38Δ::kanMX CFP-atg8::leu1+* | Figure 1B |
| DY44351 | h? | *leu1-32 atg38-YFH::leu1+ CFP-atg8::leu1+* | Figure 1C |
| DY44352 | h? | *leu1-32 atg14Δ::kanMX atg38-YFH::leu1+ CFP-atg8::leu1+* | Figure 1C |
| DY37952 | h+ | *leu1-32::nmt1p-atg38-mCherry(leu1+)* | Figure 1E and 1F |
| DY37960 | h+ | *leu1-32::nmt1p-atg38(Δ176-181)-mCherry(leu1+)* | Figure 1E |
| DY37954 | h+ | *leu1-32::nmt1p-atg38^F178A^-mCherry(leu1+)* | Figure 1E |
| DY37956 | h+ | *leu1-32::nmt1p-atg38^V181A^-mCherry(leu1+)* | Figure 1E |
| DY37958 | h+ | *leu1-32::nmt1p-atg38^F178A,V181A^-mCherry(leu1+)* | Figure 1E |
| DY37950 | h+ | *ura4-D18 leu1-32::pDUAL-vector(leu1+) ars1::41nmt1p-GFP-atg8(ura4+)* | Figure 1E and 1F |
| DY37933 | h? | *ura4-D18 leu1-32::nmt1p-atg38-mCherry(leu1+) ars1::41nmt1p-GFP-atg8(ura4+)* | Figure 1E and 1F |
| DY37944 | h? | *ura4-D18 leu1-32::nmt1p-atg38(Δ176-181)-mCherry(leu1+) ars1::41nmt1p-GFP-atg8(ura4+)* | Figure 1E |
| DY37936 | h? | *ura4-D18 leu1-32::nmt1p-atg38^F178A^-mCherry(leu1+) ars1::41nmt1p-GFP-atg8(ura4+)* | Figure 1E |
| DY37939 | h? | *ura4-D18 leu1-32::nmt1p-atg38^V181A^-mCherry(leu1+) ars1::41nmt1p-GFP-atg8(ura4+)* | Figure 1E |
| DY37941 | h? | *ura4-D18 leu1-32::nmt1p-atg38^F178A,V181A^-mCherry(leu1+) ars1::41nmt1p-GFP-atg8(ura4+)* | Figure 1E |
| DY41396 | h+ | *ura4-D18 leu1-32::pDUAL-vector(leu1+) ars1::41nmt1p-GFP-atg8^P52A,R67A^(ura4+)* | Figure 1F |
| DY42065 | h+ | *ura4-D18 leu1-32::nmt1p-atg38-mCherry(leu1+) ars1::41nmt1p-GFP-atg8^P52A,R67A^(ura4+)* | Figure 1F |
| DY38206 | h+ | *ura4-D18 pho8Δ::natMX leu1-32::pDUAL-vector(leu1+) ars1::41nmt1p-pho8Δ60(S. cerevisiae)-GFP(ura4+)* | Figure 2A, 7A, S3A, and S7B |
| DY38336 | h? | *ura4-D18 pho8Δ::natMX atg38Δ::kanMX leu1-32::pDUAL-vector(leu1+) ars1::41nmt1p-pho8Δ60(S.cerevisiae)-GFP(ura4+)* | Figure 2A and 7A |
| DY38858 | h? | *ura4-D18 pho8Δ::natMX atg38Δ::kanMX leu1-32::atg38p-13MYC-atg38(leu1+) ars1::41nmt1p-pho8Δ60(S. cerevisiae)-GFP(ura4+)* | Figure 2A |
| DY38717 | h? | *ura4-D18 pho8Δ::natMX atg38Δ::kanMX leu1-32::atg38p-13MYC-atg38^F178A,V181A^(leu1+) ars1::41nmt1p-pho8Δ60(S. cerevisiae)-GFP(ura4+)* | Figure 2A |
| DY39197 | h? | *ura4-D18 pho8Δ::natMX atg38Δ::kanMX leu1-32::atg38p-13MYC-atg38(1-181)^F178A,V181A^-3×EEEWEEL-atg38(182-424)(leu1+) ars1::41nmt1p-pho8Δ60(S. cerevisiae)-GFP(ura4+)* | Figure 2A |
| DY38259 | h− | *ura4-D18 leu1-32::pDUAL-vector(leu1+) ars1::nmt1p-tdh1-YFH(ura4+)* | Figure 2B and S7A |
| DY38265 | h− | *ura4-D18 atg38Δ::kanMX leu1-32::pDUAL-vector(leu1+) ars1::nmt1p-tdh1-YFH(ura4+)* | Figure 2B, 2C, and S7A |
| DY38689 | h+ | *ura4-D18 atg38Δ::kanMX leu1-32::atg38p-13MYC-atg38(leu1+) ars1::nmt1p-tdh1-YFH(ura4+)* | Figure 2B and 2C |
| DY38701 | h+ | *ura4-D18 atg38Δ::kanMX leu1-32::atg38p-13MYC-atg38^F178A,V181A^(leu1+) ars1::nmt1p-tdh1-YFH(ura4+)* | Figure 2B and 2C |
| DY39189 | h+ | *ura4-D18 atg38Δ::kanMX leu1-32::atg38p-13MYC-atg38(1-181)^F178A,V181A^-3×EEEWEEL-atg38(182-424)(leu1+) ars1::nmt1p-tdh1-YFH(ura4+)* | Figure 2B and 2C |
| DY41662 | h? | *leu1-32 atg1Δ::natMX atg38Δ::hphMX atg38p-atg38-CFP::kanMX atg13-YFH::leu1+* | Figure 3A, 3B, and S4B |
| DY41664 | h? | *leu1-32 atg1Δ::natMX atg38Δ::hphMX atg38p-atg38^F178A,V181A^-CFP::kanMX atg13-YFH::leu1+* | Figure 3A, 3B, and S4B |
| DY41670 | h− | *leu1-32 atg1Δ::natMX atg38Δ::hphMX atg9-YFP::bsdMX atg38p-atg38-CFP::kanMX* | Figure 3A, 3B, and S4B |
| DY41672 | h− | *leu1-32 atg1Δ::natMX atg38Δ::hphMX atg9-YFP::bsdMX atg38p-atg38^F178A,V181A^-CFP::kanMX* | Figure 3A, 3B, and S4B |
| DY43054 | h? | *leu1-32 atg1Δ::natMX atg38Δ::hphMX atg38p-atg38-CFP::kanMX atg14-YFH::leu1+* | Figure 3A, 3B, S4B, and S7C |
| DY43048 | h? | *leu1-32 atg1Δ::natMX atg38Δ::hphMX atg38p-atg38^F178A,V181A^-CFP::kanMX atg14-YFH::leu1+* | Figure 3A, 3B, and S4B |
| DY41814 | h? | *leu1-32 atg1Δ::natMX atg38Δ::hphMX atg38p-atg38-CFP::kanMX atg2-YFH::leu1+* | Figure 3A, 3B, S4B, and S7C |
| DY41816 | h? | *leu1-32 atg1Δ::natMX atg38Δ::hphMX atg38p-atg38^F178A,V181A^-CFP::kanMX atg2-YFH::leu1+* | Figure 3A, 3B, and S4B |
| DY41542 | h? | *leu1-32 atg1Δ::natMX atg38Δ::hphMX atg38p-atg38-CFP::kanMX atg18b-YFH::leu1+* | Figure 3A, 3B, S4B, and S7C |
| DY41544 | h? | *leu1-32 atg1Δ::natMX atg38Δ::hphMX atg38p-atg38^F178A,V181A^-CFP::kanMX atg18b-YFH::leu1+* | Figure 3A, 3B, and S4B |
| DY41538 | h? | *leu1-32 atg1Δ::natMX atg38Δ::hphMX atg38p-atg38-CFP::kanMX atg24b-YFH::leu1+* | Figure 3A, 3B, S4B, and S7C |
| DY41540 | h? | *leu1-32 atg1Δ::natMX atg38Δ::hphMX atg38p-atg38^F178A,V181A^-CFP::kanMX atg24b-YFH::leu1+* | Figure 3A, 3B, and S4B |
| DY41384 | h? | *leu1-32 atg1Δ::natMX atg38Δ::hphMX atg38p-atg38-CFP::kanMX atg5-YFH::leu1+* | Figure 3A, 3B, S4B, and S7C |
| DY41386 | h? | *leu1-32 atg1Δ::natMX atg38Δ::hphMX atg38p-atg38^F178A,V181A^-CFP::kanMX atg5-YFH::leu1+* | Figure 3A, 3B, and S4B |
| DY41380 | h? | *leu1-32 atg1Δ::natMX atg38Δ::hphMX atg38p-atg38-CFP::kanMX atg16-YFH::leu1+* | Figure 3A, 3B, S4B, and S7C |
| DY41382 | h? | *leu1-32 atg1Δ::natMX atg38Δ::hphMX atg38p-atg38^F178A,V181A^-CFP::kanMX atg16-YFH::leu1+* | Figure 3A, 3B, and S4B |
| DY39878 | h− | *leu1-32 atg1Δ::natMX atg38Δ::hphMX atg38p-atg38-3×GFP::kanMX CFP-Atg8::leu1+* | Figure 3A, 3B, S4B, and S7C |
| DY39882 | h? | *leu1-32 atg1Δ::natMX atg38Δ::hphMX atg38p-atg38^F178A,V181A^-3×GFP::kanMX CFP-Atg8::leu1+* | Figure 3A, 3B, and S4B |
| DY38491 | h? | *ura4-D18 fsc1Δ::natMX cpy1-mCherry::hphMX leu1-32::pDUAL-vector(leu1+) ars1::nmt1p-tdh1-YFH(ura4+)* | Figure 4A, 4B, 4C, 4D, 7B, 7C, 7D, and 7E |
| DY38489 | h? | *ura4-D18 fsc1Δ::natMX atg38Δ::kanMX cpy1-mCherry::hphMX leu1-32::pDUAL-vector(leu1+) ars1::nmt1p-tdh1-YFH(ura4+)* | Figure 4A and 4C |
| DY38704 | h? | *ura4-D18 fsc1Δ::natMX atg38Δ::kanMX cpy1-mCherry::hphMX leu1-32::atg38p-13MYC-atg38(leu1+) ars1::nmt1p-tdh1-YFH(ura4+)* | Figure 4A, 4B, 4C, and 4D |
| DY38714 | h? | *ura4-D18 fsc1Δ::natMX atg38Δ::kanMX cpy1-mCherry::hphMX leu1-32::atg38p-13MYC-atg38^F178A,V181A^(leu1+) ars1::nmt1p-tdh1-YFH(ura4+)* | Figure 4A, 4B, 4C, and 4D |
| DY39677 | h? | *ura4-D18 fsc1Δ::natMX atg38Δ::kanMX cpy1-mCherry::hphMX leu1-32::atg38p-13MYC-atg38(1-181)^F178A,V181A^-3×EEEWEEL-atg38(182-424)(leu1+) ars1::nmt1p-tdh1-YFH(ura4+)* | Figure 4A, 4B, 4C, and 4D |
| DY42288 | h? | *leu1-32 atg1Δ::natMX atg38Δ::hphMX atg38p-atg38-3×GFP::kanMX Atg17-mCherry::bsdMX CFP-Atg8::leu1+* | Figure 5A and 5B |
| DY42402 | h? | *leu1-32 atg1Δ::natMX atg38Δ::hphMX atg38p-atg38^F178A,V181A^-3×GFP::kanMX Atg17-mCherry::bsdMX CFP-Atg8::leu1+* | Figure 5A and 5B |
| DY42279 | h? | *leu1-32 atg1Δ::natMX atg38p-atg38-3×GFP::kanMX Atg17-mCherry::bsdMX CFP-Atg8::leu1+* | Figure 5C and 5D |
| DY42286 | h? | *leu1-32 atg1Δ::natMX atg38p-atg38^F178A,V181A^-3×GFP::kanMX Atg17-mCherry::bsdMX CFP-Atg8::leu1+* | Figure 5C and 5D |
| DY42289 | h? | *atg1Δ::natMX atg38p-atg38-3×GFP::kanMX atg17-mCherry::bsdMX leu1-32::atg8p-CFP-atg8(leu1+)* | Figure 5E and 5F |
| DY42406 | h? | *atg1Δ::natMX atg38p-atg38-3×GFP::kanMX atg17-mCherry::bsdMX leu1-32::atg8p-CFP-atg8^P52A,R67A^(leu1+)* | Figure 5E and 5F |
| DY43040 | h− | *leu1-32 atg1Δ::natMX atg38Δ::hphMX atg38p-atg38-3×GFP::kanMX atg17-mCherry::bsdMX CFP-atg8::leu1+* | Figure 6A and 6B |
| DY43044 | h? | *leu1-32 atg1Δ::natMX atg18aΔ::kanMX atg38Δ::hphMX atg38p-atg38-3×GFP::kanMX atg17-mCherry::bsdMX CFP-atg8::leu1+* | Figure 6A and 6B |
| DY43042 | h? | *leu1-32 atg1Δ::natMX atg5Δ::kanMX atg38Δ::hphMX atg38p-atg38-3×GFP::kanMX atg17-mCherry::bsdMX CFP-atg8::leu1+* | Figure 6A and 6B |
| DY43046 | h? | *leu1-32 atg1Δ::natMX atg16Δ::kanMX atg38Δ::hphMX atg38p-atg38-3×GFP::kanMX atg17-mCherry::bsdMX CFP-atg8::leu1+* | Figure 6A and 6B |
| DY42711 | h− | *leu1-32 atg1Δ::natMX Atg17-mCherry::bsdMX atg14-YFH::leu1+* | Figure 6C, 6D, and S6B |
| DY42974 | h? | *leu1-32 atg1Δ::natMX atg18aΔ::kanMX Atg17-mCherry::bsdMX atg14-YFH::leu1+* | Figure 6C, 6D, and S6B |
| DY42968 | h? | *leu1-32 atg1Δ::natMX atg5Δ::kanMX Atg17-mCherry::bsdMX atg14-YFH::leu1+* | Figure 6C, 6D, and S6B |
| DY42970 | h? | *leu1-32 atg1Δ::natMX atg16Δ::kanMX Atg17-mCherry::bsdMX atg14-YFH::leu1+* | Figure 6C, 6D, and S6B |
| DY43402 | h− | *atg1Δ::natMX atg8Δ::kanMX atg38p-atg38-3×GFP::kanMX atg17-mCherry::bsdMX leu1-32::atg8p-CFP-atg8(leu1+)* | Figure 6E and 6F |
| DY43400 | h− | *atg1Δ::natMX atg8Δ::kanMX atg38p-atg38-3×GFP::kanMX atg17-mCherry::bsdMX leu1-32::atg8p-CFP(leu1+)* | Figure 6E and 6F |
| DY43404 | h− | *atg1Δ::natMX atg8Δ::kanMX atg38p-atg38-3×GFP::kanMX atg17-mCherry::bsdMX leu1-32::atg8p-CFP-atg8^P52A,R67A^(leu1+)* | Figure 6E and 6F |
| DY43406 | h− | *atg1Δ::natMX atg8Δ::kanMX atg38p-atg38-3×GFP::kanMX atg17-mCherry::bsdMX leu1-32::atg8p-CFP-atg8(1-115)(leu1+)* | Figure 6E and 6F |
| DY39833 | h? | *ura4-D18 pho8Δ::natMX atg38Δ::hphMX leu1-32::atg38p-13MYC-atg38^F178A,V181A^(leu1+) ars1::41nmt1p-pho8Δ60(S.cerevisiae)-GFP(ura4+)* | Figure 7A |
| DY40341 | h? | *ura4-D18 pho8Δ::natMX atg38Δ::hphMX 41nmt1p-mCherry-atg14::kanMX leu1-32::atg38p-13MYC-atg38^F178A,V181A^(leu1+) ars1::41nmt1p-pho8Δ60(S.cerevisiae)-GFP(ura4+)* | Figure 7A |
| DY40335 | h? | *ura4-D18 pho8Δ::natMX atg38Δ::hphMX 41nmt1p-mCherry-3×EEEWEEL-atg14::kanMX leu1-32::atg38p-13MYC-atg38^F178A,V181A^(leu1+) ars1::41nmt1p-pho8Δ60(S. cerevisiae)-GFP(ura4+)* | Figure 7A |
| DY40338 | h? | *ura4-D18 pho8Δ::natMX atg38Δ::hphMX 41nmt1p-mCherry-atg14-3×EEEWEEL::kanMX leu1-32::atg38p-13MYC-atg38^F178A,V181A^(leu1+) ars1::41nmt1p-pho8Δ60(S. cerevisiae)-GFP(ura4+)* | Figure 7A |
| DY40448 | h? | *ura4-D18 fsc1Δ::natMX atg38Δ::bsdMX cpy1-mcherry::hphMX 41nmt1p-mCherry-atg14::kanMX leu1-32::atg38p-13MYC-atg38^F178A,V181A^(leu1+) ars1::nmt1p-tdh1-YFH(ura4+)* | Figure 7B, 7C, 7D, and 7E |
| DY40443 | h? | *ura4-D18 fsc1Δ::natMX atg38Δ::bsdMX cpy1-mcherry::hphMX 41nmt1p-mCherry-3×EEEWEEL-atg14::kanMX leu1-32::atg38p-13MYC-atg38^F178A,V181A^(leu1+) ars1::nmt1p-tdh1-YFH(ura4+)* | Figure 7B, 7C, 7D, and 7E |
| DY40445 | h? | *ura4-D18 fsc1Δ::natMX atg38Δ::bsdMX cpy1-mcherry::hphMX 41nmt1p-mCherry-atg14-3×EEEWEEL::kanMX leu1-32::atg38p-13MYC-atg38^F178A,V181A^(leu1+) ars1::nmt1p-tdh1-YFH(ura4+)* | Figure 7B, 7C, 7D, and 7E |
| DY11900 | h− | *leu1-32 atg1Δ::kanMX CFP-atg8::leu1+* | Figure S1A |
| DY11930 | h− | *leu1-32 atg10Δ::kanMX CFP-atg8::leu1+* | Figure S1A |
| DY11932 | h? | *leu1-32 atg16Δ::kanMX CFP-atg8::leu1+* | Figure S1A |
| DY44353 | h? | *leu1-32 isp6Δ::kanMX CFP-atg8::leu1+* | Figure S1A |
| DY44354 | h? | *leu1-32 isp6Δ::natMX atg1Δ::kanMX CFP-atg8::leu1+* | Figure S1A |
| DY44355 | h? | *leu1-32 isp6Δ::natMX atg10Δ::kanMX CFP-atg8::leu1+* | Figure S1A |
| DY44356 | h? | *leu1-32 isp6Δ::natMX atg14Δ::kanMX CFP-atg8::leu1+* | Figure S1A |
| DY44357 | h? | *leu1-32 isp6Δ::natMX atg16Δ::kanMX CFP-atg8::leu1+* | Figure S1A |
| DY44358 | h? | *isp6Δ::kanMX arg6::PB(ura4+)(TTAA at chromosome 2 coordinate 1229486) leu1-32::nmt1p-PBase(leu1+)* | Figure S1C |
| DY3957 | h+ | *his3-D1 atg6-YFH::leu1+* | Figure S1D |
| DY3961 | h+ | *his3-D1 atg14-YFH::leu1+* | Figure S1E |
| DY45438 | h− | *his3-D1 atg38Δ::kanMX leu1-32::nmt1p-GFP-atg38(leu1+)* | Figure S1F |
| DY44359 | h− | *cpy1-mCherry::hphMX leu1-32::nmt1p-tdh1-YFH(leu1+) CFP-atg8::leu1+* | Figure S2A, S2B, and S2C |
| DY44360 | h? | *atg14Δ::kanMX cpy1-mCherry::hphMX leu1-32::nmt1p-tdh1-YFH(leu1+) CFP-atg8::leu1+* | Figure S2A, S2B, and S2C |
| DY44361 | h? | *atg38Δ::kanMX cpy1-mCherry::hphMX leu1-32::nmt1p-tdh1-YFH(leu1+) CFP-atg8::leu1+* | Figure S2A, S2B, and S2C |
| DY44362 | h? | *atg6Δ::kanMX cpy1-mCherry::hphMX leu1-32::nmt1p-tdh1-YFH(leu1+) CFP-atg8::leu1+* | Figure S2A, S2B, and S2C |
| DY44363 | h? | *vps38Δ::kanMX cpy1-mCherry::hphMX leu1-32::nmt1p-tdh1-YFH(leu1+) CFP-atg8::leu1+* | Figure S2A, S2B, and S2C |
| DY44364 | h? | *vps34Δ::kanMX cpy1-mCherry::hphMX leu1-32::nmt1p-tdh1-YFH(leu1+) CFP-atg8::leu1+* | Figure S2A, S2B, and S2C |
| DY44365 | h? | *vps15Δ::kanMX cpy1-mCherry::hphMX leu1-32::nmt1p-tdh1-YFH(leu1+) CFP-atg8::leu1+* | Figure S2A, S2B, and S2C |
| DY40076 | h− | *ura4-D18 pho8Δ::natMX atg8Δ::kanMX leu1-32::pDUAL-vector(leu1+) ars1::41nmt1p-pho8Δ60(S. cerevisiae)-GFP(ura4+)* | Figure S3A |
| DY40078 | h− | *ura4-D18 pho8Δ::natMX atg8Δ::kanMX leu1-32::atg8p-GFP-atg8(leu1+) ars1::41nmt1p-pho8Δ60(S. cerevisiae)-GFP(ura4+)* | Figure S3A |
| DY40081 | h− | *ura4-D18 pho8Δ::natMX atg8Δ::kanMX leu1-32::atg8p-GFP-atg8^P52A,R67A^(leu1+) ars1::41nmt1p-pho8Δ60(S. cerevisiae)-GFP(ura4+)* | Figure S3A |
| DY40625 | h? | *ura4-D18 fsc1Δ::natMX atg8Δ::kanMX cpy1-mCherry::hphMX leu1-32::atg8p-CFP(leu1+) ars1::nmt1p-tdh1-YFH(ura4+)* | Figure S3B |
| DY40627 | h? | *ura4-D18 fsc1Δ::natMX atg8Δ::kanMX cpy1-mCherry::hphMX leu1-32::atg8p-CFP-atg8(leu1+) ars1::nmt1p-tdh1-YFH(ura4+)* | Figure S3B and S3C |
| DY40630 | h? | *ura4-D18 fsc1Δ::natMX atg8Δ::kanMX cpy1-mCherry::hphMX leu1-32::atg8p-CFP-atg8^P52A,R67A^(leu1+) ars1::nmt1p-tdh1-YFH(ura4+)* | Figure S3B and S3C |
| DY44829 | h? | *leu1-32 atg38Δ::hphMX atg38p-atg38-CFP::kanMX atg13-YFH::leu1+* | Figure S4A |
| DY44832 | h? | *leu1-32 atg38Δ::hphMX atg38p-atg38^F178A,V181A^-CFP::kanMX atg13-YFH::leu1+* | Figure S4A |
| DY44835 | h? | *leu1-32 atg38Δ::hphMX atg9-YFP::bsdMX atg38p-atg38-CFP::kanMX* | Figure S4A |
| DY44838 | h? | *leu1-32 atg38Δ::hphMX atg9-YFP::bsdMX atg38p-atg38^F178A,V181A^-CFP::kanMX* | Figure S4A |
| DY44994 | h? | *leu1-32 atg38Δ::hphMX atg38-atg38p-CFP::kanMX atg14-YFH::leu1+* | Figure S4A |
| DY44996 | h? | *leu1-32 atg38Δ::hphMX atg38p-atg38^F178A,V181A^-CFP::kanMX atg14-YFH::leu1+* | Figure S4A |
| DY39751 | h? | *leu1-32 atg38Δ::hphMX atg38p-atg38-3×GFP::kanMX CFP-Atg8::leu1+* | Figure S4A |
| DY39754 | h? | *leu1-32 atg38Δ::hphMX atg38p-atg38^F178A,V181A^-3×GFP::kanMX CFP-Atg8::leu1+* | Figure S4A |
| DY44998 | h? | *leu1-32 atg38Δ::hphMX atg38p-atg38-CFP::kanMX atg2-YFH::leu1+* | Figure S4A |
| DY45000 | h? | *leu1-32 atg38Δ::hphMX atg38p-atg38^F178A,V181A^-CFP::kanMX atg2-YFH::leu1+* | Figure S4A |
| DY45002 | h? | *leu1-32 atg38Δ::hphMX atg38p-atg38-CFP::kanMX atg18b-YFH::leu1+* | Figure S4A |
| DY45004 | h? | *leu1-32 atg38Δ::hphMX atg38p-atg38^F178A,V181A^-CFP::kanMX atg18b-YFH::leu1+* | Figure S4A |
| DY45006 | h? | *leu1-32 atg38Δ::hphMX atg38p-atg38-CFP::kanMX atg24b-YFH::leu1+* | Figure S4A |
| DY45008 | h? | *leu1-32 atg38Δ::hphMX atg38p-atg38^F178A,V181A^-CFP::kanMX atg24b-YFH::leu1+* | Figure S4A |
| DY45010 | h? | *leu1-32 atg38Δ::hphMX atg38p-atg38-CFP::kanMX atg5-YFH::leu1+* | Figure S4A |
| DY45012 | h? | *leu1-32 atg38Δ::hphMX atg38p-atg38^F178A,V181A^-CFP::kanMX atg5-YFH::leu1+* | Figure S4A |
| DY45014 | h? | *leu1-32 atg38Δ::hphMX atg38p-atg38-CFP::kanMX atg16-YFH::leu1+* | Figure S4A |
| DY45016 | h? | *leu1-32 atg38Δ::hphMX atg38p-atg38^F178A,V181A^-CFP::kanMX atg16-YFH::leu1+* | Figure S4A |
| DY41274 | h− | *leu1-32 atg1Δ::natMX atg38Δ::hphMX atg13-YFH::leu1+* | Figure S4B |
| DY41408 | h− | *leu1-32 atg1Δ::natMX atg38Δ::hphMX atg9-YFP::bsdMX* | Figure S4B |
| DY41302 | h? | *leu1-32 atg1Δ::natMX atg38Δ::hphMX atg14-YFH::leu1+* | Figure S4B |
| DY44875 | h? | *leu1-32 atg1Δ::natMX atg38Δ::hphMX atg38p-atg38(1-181)^F178A,V181A^-3×EEEWEEL-atg38(182-424)-CFP::kanMX atg14-YFH::leu1+* | Figure S4B |
| DY41557 | h− | *leu1-32 atg1Δ::natMX atg38Δ::hphMX atg2-YFH::leu1+* | Figure S4B |
| DY44877 | h− | *leu1-32 atg1Δ::natMX atg38Δ::hphMX atg38p-atg38(1-181)^F178A,V181A^-3×EEEWEEL-atg38(182-424)-CFP::kanMX atg2-YFH::leu1+* | Figure S4B |
| DY41289 | h+ | *leu1-32 atg1Δ::natMX atg38Δ::hphMX atg18b-YFH::leu1+* | Figure S4B |
| DY44879 | h+ | *leu1-32 atg1Δ::natMX atg38Δ::hphMX atg38p-atg38(1-181)^F178A,V181A^-3×EEEWEEL-atg38(182-424)-CFP::kanMX atg18b-YFH::leu1+* | Figure S4B |
| DY41286 | h+ | *leu1-32 atg1Δ::natMX atg38Δ::hphMX atg24b-YFH::leu1+* | Figure S4B |
| DY44881 | h+ | *leu1-32 atg1Δ::natMX atg38Δ::hphMX atg38p-atg38(1-181)^F178A,V181A^-3×EEEWEEL-atg38(182-424)-CFP::kanMX atg24b-YFH::leu1+* | Figure S4B |
| DY41250 | h− | *leu1-32 atg1Δ::natMX atg38Δ::hphMX atg5-YFH::leu1+* | Figure S4B |
| DY44883 | h− | *leu1-32 atg1Δ::natMX atg38Δ::hphMX atg38p-atg38(1-181)^F178A,V181A^-3×EEEWEEL-atg38(182-424)-CFP::kanMX atg5-YFH::leu1+* | Figure S4B |
| DY41244 | h+ | *leu1-32 atg1Δ::natMX atg38Δ::hphMX atg16-YFH::leu1+* | Figure S4B |
| DY44885 | h+ | *leu1-32 atg1Δ::natMX atg38Δ::hphMX atg38p-atg38(1-181)^F178A,V181A^-3×EEEWEEL-atg38(182-424)-CFP::kanMX atg16-YFH::leu1+* | Figure S4B |
| DY40357 | h+ | *leu1-32 atg1Δ::natMX atg38Δ::hphMX CFP-atg8::leu1+* | Figure S4B |
| DY44905 | h+ | *leu1-32 atg1Δ::natMX atg38Δ::hphMX atg38p-atg38(1-181)^F178A,V181A^-3×EEEWEEL-atg38(182-424)-CFP::kanMX CFP-atg8::leu1+* | Figure S4B |
| DY37337 | h+ | *leu1-32::pDUAL-vector(leu1+)* | Figure S5A, S5B, S6A, and S6B |
| DY40624 | h− | *leu1-32 atg1Δ::natMX atg38Δ::hphMX atg38p-atg38-3×GFP::kanMX* | Figure S5A and S6A |
| DY42686 | h+ | *leu1-32 atg1Δ::natMX atg38Δ::hphMX atg38p-atg38^F178A,V181A^-3×GFP::kanMX* | Figure S5A |
| DY42975 | h? | *atg1Δ::natMX atg8Δ::kanMX leu1-32::atg8p-CFP-atg8(leu1+)* | Figure S5B |
| DY42976 | h? | *atg1Δ::natMX atg8Δ::kanMX leu1-32:Patg8p-CFP-atg8^P52A,R67A^(leu1+)* | Figure S5B |
| DY45029 | h? | *atg1Δ::natMX atg8Δ::hphMX leu1-32::atg8p-CFP-atg8(leu1+)* | Figure S5C |
| DY45032 | h? | *atg1Δ::natMX atg18aΔ::kanMX atg8Δ::hphMX leu1-32::atg8p-CFP-atg8(leu1+)* | Figure S5C |
| DY45038 | h? | *atg1Δ::natMX atg16Δ::kanMX atg8Δ::hphMX leu1-32::atg8p-CFP-atg8(leu1+)* | Figure S5C |
| DY45052 | h? | *ura4-D18 atg1Δ::natMX atg38Δ::kanMX atg8Δ::hphMX leu1-32::atg8p-CFP-atg8(leu1+) ars1::atg38p-13MYC-atg38^F178A,V181A^(ura4+)* | Figure S5C |
| DY45058 | h? | *ura4-D18 atg1Δ::natMX atg8Δ::hphMX leu1-32::atg8p-CFP-atg8(leu1+) ars1::atg38p-13MYC-atg38^F178A,V181A^(ura4+)* | Figure S5C |
| DY45434 | h? | *atg1Δ::natMX atg8Δ::hphMX leu1-32::atg8p-CFP-atg8^P52A,R67A^(leu1+)* | Figure S5C |
| DY45436 | h? | *atg1Δ::natMX leu1-32::atg8p-CFP-atg8^P52A,R67A^(leu1+)* | Figure S5C |
| DY45077 | h? | *atg1Δ::natMX leu1-32::atg8p-CFP-atg8(leu1+)* | Figure S5C |
| DY42693 | h? | *leu1-32 atg1Δ::natMX atg18aΔ::kanMX atg38Δ::hphMX atg38p-atg38-3×GFP::kanMX* | Figure S6A |
| DY42703 | h? | *leu1-32 atg1Δ::natMX atg5Δ::natMX atg38Δ::hphMX atg38p-atg38-3×GFP::kanMX* | Figure S6A |
| DY42701 | h? | *leu1-32 atg1Δ::natMX atg16Δ::kanMX atg38Δ::hphMX atg38p-atg38-3×GFP::kanMX* | Figure S6A |
| DY40319 | h+ | *ura4-D18 atg38Δ::hphMX leu1-32::atg38p-13MYC-atg38^F178A,V181A^(leu1+) ars1::nmt1p-tdh1-YFH(ura4+)* | Figure S7A |
| DY40434 | h+ | *ura4-D18 atg38Δ::hphMX 41nmt1p-mCherry-atg14::kanMX leu1-32::atg38p-13MYC-atg38^F178A,V181A^(leu1+) ars1::nmt1p-tdh1-YFH(ura4+)* | Figure S7A |
| DY40428 | h+ | *ura4-D18 atg38Δ::hphMX 41nmt1p-mCherry-3×EEEWEEL-atg14::kanMX leu1-32::atg38p-13MYC-atg38^F178A,V181A^(leu1+) ars1::nmt1p-tdh1-YFH(ura4+)* | Figure S7A |
| DY40431 | h+ | *ura4-D18 atg38Δ::hphMX 41nmt1p-mCherry-atg14-3×EEEWEEL::kanMX leu1-32::atg38p-13MYC-atg38^F178A,V181A^(leu1+) ars1::nmt1p-tdh1-YFH(ura4+)* | Figure S7A |
| DY44473 | h+ | *ura4-D18 pho8Δ::natMX leu1-32::41nmt1p-mCherry-3×EEEWEEL-atg14(leu1+) ars1::41nmt1p-pho8Δ60(S. cerevisiae)-GFP(ura4+)* | Figure S7B |
| DY40028 | h? | *ura4-D18 pho8Δ::natMX atg38Δ::kanMX leu1-32::41nmt1p-mCherry-3×EEEWEEL-atg14(leu1+) ars1::41nmt1p-pho8Δ60(S. cerevisiae)-GFP(ura4+)* | Figure S7B |
| DY44470 | h+ | *ura4-D18 pho8Δ::natMX leu1-32::41nmt1p-mCherry-atg14-3×EEEWEEL(leu1+) ars1::41nmt1p-pho8Δ60(S. cerevisiae)-GFP(ura4+)* | Figure S7B |
| DY40025 | h? | *ura4-D18 pho8Δ::natMX atg38Δ::kanMX leu1-32::41nmt1p-mCherry-atg14-3×EEEWEEL(leu1+) ars1::41nmt1p-pho8Δ60(S. cerevisiae)-GFP(ura4+)* | Figure S7B |
| DY44988 | h? | *ura4-D18 atg1Δ::natMX atg38Δ::hphMX atg38p-atg38^F178A,V181A^-CFP::kanMX atg14-YFH::leu1+ ars1::41nmt1p-mCherry-atg14(ura4+)* | Figure S7C |
| DY44990 | h? | *ura4-D18 atg1Δ::natMX atg38Δ::hphMX atg38p-atg38^F178A,V181A^-CFP::kanMX atg14-YFH::leu1+ ars1::41nmt1p-mCherry-3×EEEWEEL-atg14(ura4+)* | Figure S7C |
| DY44992 | h? | *ura4-D18 atg1Δ::natMX atg38Δ::hphMX atg38p-atg38^F178A,V181A^-CFP::kanMX atg14-YFH::leu1+ ars1::41nmt1p-mCherry-atg14-3×EEEWEEL(ura4+)* | Figure S7C |
| DY44965 | h? | *ura4-D18 atg1Δ::natMX atg38Δ::hphMX atg38p-atg38^F178A,V181A^-CFP::kanMX atg2-YFH::leu1+ ars1::41nmt1p-mCherry-atg14(ura4+)* | Figure S7C |
| DY44967 | h? | *ura4-D18 atg1Δ::natMX atg38Δ::hphMX atg38p-atg38^F178A,V181A^-CFP::kanMX atg2-YFH::leu1+ ars1::41nmt1p-mCherry-3×EEEWEEL-atg14(ura4+)* | Figure S7C |
| DY44969 | h? | *ura4-D18 atg1Δ::natMX atg38Δ::hphMX atg38p-atg38^F178A,V181A^-CFP::kanMX atg2-YFH::leu1+ ars1::41nmt1p-mCherry-atg14-3×EEEWEEL(ura4+)* | Figure S7C |
| DY44983 | h? | *ura4-D18 atg1Δ::natMX atg38Δ::hphMX atg38p-atg38^F178A,V181A^-CFP::kanMX atg18b-YFH::leu1+ ars1::41nmt1p-mCherry-atg14(ura4+)* | Figure S7C |
| DY44984 | h? | *ura4-D18 atg1Δ::natMX atg38Δ::hphMX atg38p-atg38^F178A,V181A^-CFP::kanMX atg18b-YFH::leu1+ ars1::41nmt1p-mCherry-3×EEEWEEL-atg14(ura4+)* | Figure S7C |
| DY44986 | h? | *ura4-D18 atg1Δ::natMX atg38Δ::hphMX atg38p-atg38^F178A,V181A^-CFP::kanMX atg18b-YFH::leu1+ ars1::41nmt1p-mCherry-atg14-3×EEEWEEL(ura4+)* | Figure S7C |
| DY44971 | h? | *ura4-D18 atg1Δ::natMX atg38Δ::hphMX atg38p-atg38^F178A,V181A^-CFP::kanMX atg24b-YFH::leu1+ ars1::41nmt1p-mCherry-atg14(ura4+)* | Figure S7C |
| DY44973 | h? | *ura4-D18 atg1Δ::natMX atg38Δ::hphMX atg38p-atg38^F178A,V181A^-CFP::kanMX atg24b-YFH::leu1+ ars1::41nmt1p-mCherry-3×EEEWEEL-atg14(ura4+)* | Figure S7C |
| DY44975 | h? | *ura4-D18 atg1Δ::natMX atg38Δ::hphMX atg38p-atg38^F178A,V181A^-CFP::kanMX atg24b-YFH::leu1+ ars1::41nmt1p-mCherry-atg14-3×EEEWEEL(ura4+)* | Figure S7C |
| DY44953 | h? | *ura4-D18 atg1Δ::natMX atg38Δ::hphMX atg38p-atg38^F178A,V181A^-CFP::kanMX atg5-YFH::leu1+ ars1::41nmt1p-mCherry-atg14(ura4+)* | Figure S7C |
| DY44955 | h? | *ura4-D18 atg1Δ::natMX atg38Δ::hphMX atg38p-atg38^F178A,V181A^-CFP::kanMX atg5-YFH::leu1+ ars1::41nmt1p-mCherry-3×EEEWEEL-atg14(ura4+)* | Figure S7C |
| DY44957 | h? | *ura4-D18 atg1Δ::natMX atg38Δ::hphMX atg38p-atg38^F178A,V181A^-CFP::kanMX atg5-YFH::leu1+ ars1::41nmt1p-mCherry-atg14-3×EEEWEEL(ura4+)* | Figure S7C |
| DY44977 | h? | *ura4-D18 atg1Δ::natMX atg38Δ::hphMX atg38p-atg38^F178A,V181A^-CFP::kanMX atg16-YFH::leu1+ ars1::41nmt1p-mCherry-atg14(ura4+)* | Figure S7C |
| DY44979 | h? | *ura4-D18 atg1Δ::natMX atg38Δ::hphMX atg38p-atg38^F178A,V181A^-CFP::kanMX atg16-YFH::leu1+ ars1::41nmt1p-mCherry-3×EEEWEEL-atg14(ura4+)* | Figure S7C |
| DY44981 | h? | *ura4-D18 atg1Δ::natMX atg38Δ::hphMX atg38p-atg38^F178A,V181A^-CFP::kanMX atg16-YFH::leu1+ ars1::41nmt1p-mCherry-atg14-3×EEEWEEL(ura4+)* | Figure S7C |
| DY44959 | h? | *ura4-D18 atg1Δ::natMX atg38Δ::hphMX atg38p-atg38^F178A,V181A^-3×GFP::kanMX CFP-atg8::leu1+ ars1::41nmt1p-mCherry-atg14(ura4+)* | Figure S7C |
| DY44961 | h? | *ura4-D18 atg1Δ::natMX atg38Δ::hphMX atg38p-atg38^F178A,V181A^-3×GFP::kanMX CFP-atg8::leu1+ ars1::41nmt1p-mCherry-3×EEEWEEL-atg14(ura4+)* | Figure S7C |
| DY44963 | h? | *ura4-D18 atg1Δ::natMX atg38Δ::hphMX atg38p-atg38^F178A,V181A^-3×GFP::kanMX CFP-atg8::leu1+ ars1::41nmt1p-mCherry-atg14-3×EEEWEEL(ura4+)* | Figure S7C |

**Table S2.** Plasmids used in this study

| Name | Descriptive name | Description |
| --- | --- | --- |
| pDB4653 | pDUAL-*nmt1p*-Atg38-mCherry | pDUAL plasmid expressing Atg38-mCherry from *nmt1* promoter |
| pDB4654 | pDUAL-*nmt1p*-Atg38(Δ176-181)-mCherry | pDUAL plasmid expressing Atg38(Δ176-181)-mCherry from *nmt1* promoter |
| pDB4655 | pDUAL-*nmt1p*-Atg38^F178A^-mCherry | pDUAL plasmid expressing Atg38^F178A^-mCherry from *nmt1* promoter |
| pDB4656 | pDUAL-*nmt1p*-Atg38^V181A^-mCherry | pDUAL plasmid expressing Atg38^V181A^-mCherry from *nmt1* promoter |
| pDB4657 | pDUAL-*nmt1p*-Atg38^F178A,V181A^-mCherry | pDUAL plasmid expressing Atg38^F178A,V181A^-mCherry from *nmt1* promoter |
| pDB4658 | pDUAL-*41nmt1p*-GFP-Atg8 | pDUAL plasmid expressing GFP-Atg8 from *41nmt1* promoter |
| pDB4659 | pDUAL-*41nmt1p*-GFP-Atg8^P52A,R67A^ | pDUAL plasmid expressing GFP-Atg8^P52A,R67A^ from *41nmt1* promoter |
| pDB4660 | pGADT7-Atg38 | Y2H prey plasmid expressing Atg38 |
| pDB4661 | pGADT7-Atg38(1-212) | Y2H prey plasmid expressing Atg38(1-212) |
| pDB4662 | pGADT7-Atg38(213-424) | Y2H prey plasmid expressing Atg38(213-424) |
| pDB4663 | pGADT7-Atg38(1-72) | Y2H prey plasmid expressing Atg38(1-72) |
| pDB4664 | pGADT7-Atg38(73-212) | Y2H prey plasmid expressing Atg38(73-212) |
| pDB4665 | pGADT7-Atg38(Δ176-181) | Y2H prey plasmid expressing Atg38(Δ176-181) |
| pDB4666 | pGADT7-Atg38^F178A,V181A^ | Y2H prey plasmid expressing Atg38^F178A,V181A^ |
| pLD13 | pGADT7-Crb2(521-778) | Y2H prey plasmid expressing Crb2(521-778) |
| pDB4667 | pGBKT7-Atg8 | Y2H bait plasmid expressing Atg8 |
| pLD5 | pGBKT7-Crb2(276-778) | Y2H bait plasmid expressing Crb2(276-778) |
| pDB4668 | pET15b-HA-Atg8 | pET15b plasmid for expressing HA-Atg8 in *E. coli* |
| pDB4669 | pET15b-HA-Atg8^P52A,R67A^ | pET15b plasmid for expressing HA-Atg8^P52A,R67A^ in *E. coli* |
| pDB4670 | pETDuet-GST-Atg38(161-190) | pETDuet plasmid for expressing Atg38(161-190) in *E. coli* |
| pDB4671 | pETDuet-GST-Atg38(161-190)^F178A,V181A^ | pETDuet plasmid for expressing Atg38(161-190) with mutations of F178A,V181A in *E. coli* |
| pDB4672 | pDUAL-*41nmt1p*-Pho8Δ60(*S. cerevisiae*)-GFP | pDUAL plasmid expressing *S. cerevisiae* Pho8Δ60 C-terminally tagged with GFP from *41nmt1* promoter |
| pDB4673 | pDUAL-*atg38p*-13*MYC*-atg38 | pDUAL plasmid expressing 13MYC-Atg38 from endogenous *atg38* promoter |
| pDB4674 | pDUAL-*atg38p*-13*MYC*-Atg38^F178A,V181A^ | pDUAL plasmid expressing 13MYC-Atg38^F178A,V181A^ from endogenous *atg38* promoter |
| pDB4675 | pDUAL-*atg38p*-13MYC-Atg38(1-181)^F178A,V181A^-3×EEEWEEL-atg38(182-424) | pDUAL plasmid expressing Atg38(1-181)^F178A,V181A^-3×EEEWEEL-Atg38(182-424) from endogenous *atg38* promoter |
| pDB4676 | pFA6a-*atg38p*-Atg38-CFP-kanMX6 | pFA6a-kanMX6 plasmid expressing Atg38-CFP from endogenous *atg38* promoter |
| pDB4677 | pFA6a-*atg38p*-Atg38^F178A,V181A^-CFP-kanMX6 | pFA6a-kanMX6 plasmid expressing Atg38^F178A,V181A^-CFP from endogenous *atg38* promoter |
| pDB4678 | pFA6a-*atg38p*-Atg38-3×GFP-kanMX6 | pFA6a-kanMX6 plasmid expressing Atg38-3×GFP from endogenous *atg38* promoter |
| pDB4679 | pFA6a-*atg38p*-Atg38^F178A,V181A^-3×GFP-kanMX6 | pFA6a-kanMX6 plasmid expressing Atg38^F178A,V181A^-3×GFP from endogenous promoter |
| pDB4680 | pDUAL-*atg8p*-GFP-Atg8 | pDUAL plasmid expressing GFP-Atg8 from endogenous *atg8* promoter |
| pDB4681 | pDUAL-*atg8p*-GFP-Atg8^P52A,R67A^ | pDUAL plasmid expressing GFP-Atg8^P52A,R67A^ from endogenous *atg8* promoter |
| pDB4682 | pDUAL-*atg8p*-CFP | pDUAL plasmid expressing CFP from *atg8* promoter |
| pDB4683 | pDUAL-*atg8p*-CFP-Atg8 | pDUAL plasmid expressing CFP-Atg8 from endogenous *atg8* promoter |
| pDB4684 | pDUAL-*atg8p*-CFP-Atg8^P52A,R67A^ | pDUAL plasmid expressing CFP-Atg8^P52A,R67A^ from endogenous *atg8* promoter |
| pDB4685 | pDUAL-*atg8p*-CFP-Atg8(1-115) | pDUAL plasmid expressing CFP-Atg8(1-115) from endogenous *atg8* promoter |
| pDB4686 | pDUAL-*41nmt1p*-mCherry-Atg14-kanMX6 | pDUAL plasmid modified with a kanMX marker expressing mCherry-Atg14 from *41nmt1* promoter |
| pDB4687 | pDUAL-*41nmt1p*-mCherry-3×EEEWEEL-Atg14-kanMX6 | pDUAL plasmid modified with a kanMX marker expressing mCherry-3×EEEWEEL-Atg14 from *41nmt1* promoter |
| pDB4688 | pDUAL-*41nmt1p*-mCherry-Atg14-3×EEEWEEL-kanMX6 | pDUAL plasmid modified with a kanMX marker expressing mCherry-Atg14-3×EEEWEEL from *41nmt1* promoter |
| pDB4689 | pDUAL-*41nmt1p*-mCherry-3×EEEWEEL-Atg14 | pDUAL plasmid expressing mCherry-3×EEEWEEL-Atg14 from *41nmt1* promoter |
| pDB4690 | pDUAL-*41nmt1p*-mCherry-Atg14-3×EEEWEEL | pDUAL plasmid expressing mCherry-Atg14-3×EEEWEEL from *41nmt1* promoter |
| pDB4705 | pDUAL-*nmt1p*-GFP-Atg38 | pDUAL plasmid expressing GFP-Atg38 from *nmt1* promoter |
| pDB4706 | pFA6a-*atg38p*-Atg38(1-181)^F178A,V181A^-3×EEEWEEL-Atg38(182-424)-CFP-kanMX | pFA6a-kanMX6 plasmid expressing Atg38(1-181)^F178A,V181A^-3×EEEWEEL-Atg38(182-424)-CFP from endogenous *atg38* promoter |
| pDB4707 | pDUAL-*41nmt1p*-mCherry-Atg14 | pDUAL plasmid expressing mCherry-Atg14 from *41nmt1* promoter |
